# Cytotoxic activity of *Kingella kingae* RtxA toxin depends on post-translational acylation of lysine residues and cholesterol binding

**DOI:** 10.1038/s41426-018-0179-x

**Published:** 2018-11-07

**Authors:** Adriana Osickova, Nataliya Balashova, Jiri Masin, Miroslav Sulc, Jana Roderova, Tomas Wald, Angela C. Brown, Evan Koufos, En Hyung Chang, Alexander Giannakakis, Edward T. Lally, Radim Osicka

**Affiliations:** 1 Institute of Microbiology of the CAS, v.v.i., Prague, Czech Republic; 2 Faculty of Science, Charles University, Prague, Czech Republic; 3 Department of Pathology, School of Dental Medicine, University of Pennsylvania, Philadelphia, PA USA; 4 Department of Chemical and Biomolecular Engineering, Lehigh University, Bethlehem, PA USA; 5 Present Address: Department of Orofacial Sciences and Program in Craniofacial Biology, University of California, San Francisco, CA USA; 6 Present Address: The Department of Cell and Molecular Biology at Karolinska Institutet, Stockholm, Sweden

## Abstract

*Kingella kingae* is a member of the commensal oropharyngeal flora of young children. Improvements in detection methods have led to the recognition of *K. kingae* as an emerging pathogen that frequently causes osteoarticular infections in children and a severe form of infective endocarditis in children and adults. *Kingella kingae* secretes a membrane-damaging RTX (Repeat in ToXin) toxin, RtxA, which is implicated in the development of clinical infections. However, the mechanism by which RtxA recognizes and kills host cells is largely unexplored. To facilitate structure-function studies of RtxA, we have developed a procedure for the overproduction and purification of milligram amounts of biologically active recombinant RtxA. Mass spectrometry analysis revealed the activation of RtxA by post-translational fatty acyl modification on the lysine residues 558 and/or 689 by the fatty-acyltransferase RtxC. Acylated RtxA was toxic to various human cells in a calcium-dependent manner and possessed pore-forming activity in planar lipid bilayers. Using various biochemical and biophysical approaches, we demonstrated that cholesterol facilitates the interaction of RtxA with artificial and cell membranes. The results of analyses using RtxA mutant variants suggested that the interaction between the toxin and cholesterol occurs via two cholesterol recognition/interaction amino acid consensus motifs located in the C-terminal portion of the pore-forming domain of the toxin. Based on our observations, we conclude that the cytotoxic activity of RtxA depends on post-translational acylation of the K558 and/or K689 residues and on the toxin binding to cholesterol in the membrane.

## Introduction

*Kingella kingae* is a fastidious, facultative anaerobic, gram-negative coccobacillus of the *Neisseriaceae* family that was first isolated in 1960 by Elizabeth King^1–3^. *Kingella kingae* is a member of the commensal oropharyngeal flora of young children, and its transmission from child to child is believed to occur through close personal contact^1,4,5^. The process of colonization likely involves the adherence of *K. kingae* to respiratory epithelial cells through type IV pili^6,7^. The maximal colonization of children by *K. kingae* occurs between the ages of 6 and 36 months, peaking in the second year of life^3^. The carriage of *K. kingae* gradually decreases in older children and adults, indicating the acquisition of immunity that eradicates the bacterium from the pharynx^4,8^. Until recently, *K. kingae* was believed to be a rare cause of infection. However, improvements in culture techniques and molecular detection methods have led to the recognition of the bacterium as an important invasive pediatric pathogen^3,9^. In several reports, *K. kingae* has been recognized as a leading cause of osteomyelitis and septic arthritis in young children^10^. *Kingella kingae* can cause other invasive infections, including occult bacteremia, infective endocarditis, pneumonia, meningitis, eye infections, peritonitis, and pericarditis^1^.

Microscopy and lactic acid dehydrogenase release experiments revealed that *K. kingae* is cytotoxic to cultured respiratory epithelial cells, macrophage-like cells and synovial cells that the bacterium encounters in the host organism^11^. These cytotoxic effects have been attributed to the *K. kingae* RTX (Repeat in ToXin) cytotoxin RtxA^11^. Experiments in an infant rat model with the RtxA-deficient mutant KKNB100 revealed that RtxA is a key virulence factor of *K. kingae*^12^. The RTX locus encoding RtxA has been identified in all *K. kingae* clinical isolates, and it has been suggested as a specific diagnostic marker of *K. kingae* infections^13,14^. However, the RTX locus has recently been identified in a novel species named *Kingella negevensis*^15^, and it was demonstrated that targeting of this marker cannot distinguish between *K. kingae* and *K. negevensis* species^16^.

**Fig. 1:**
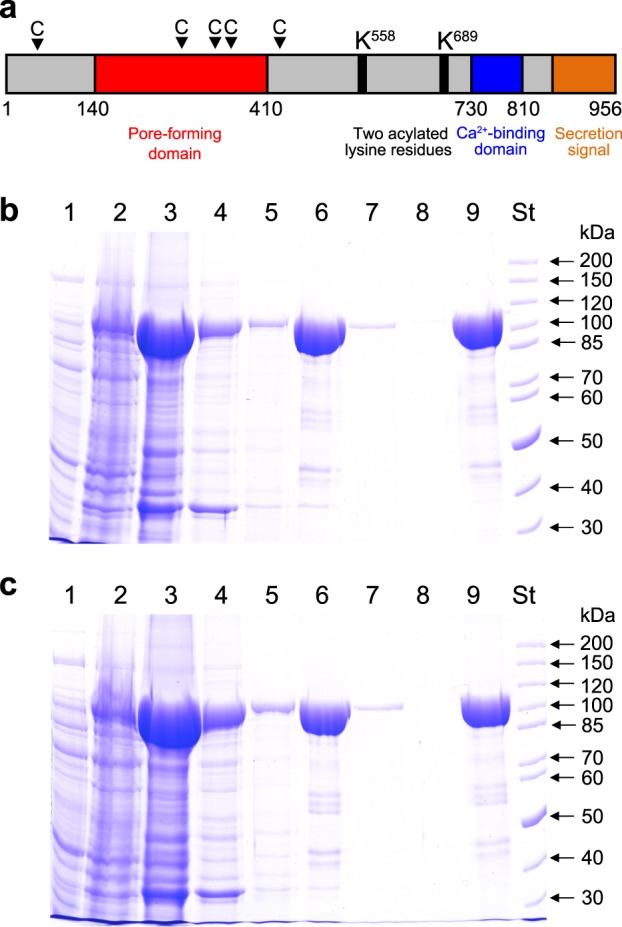
Schematic representation and purification process for RtxA and proRtxA. **a** Scheme of the *K. kingae* RtxA molecule, with several different areas predicted from homology with other RTX toxins. The arrowheads with a letter C indicate the predicted CRAC and CARC motifs. The RtxA **(b)** and proRtxA (**c**) proteins were produced in *E. coli* BL21/pMM100 cells and purified by a combination of affinity and hydrophobic chromatographies. Lanes: 1, crude extract from uninduced cells; 2, crude extract from cells induced with isopropyl β-D-1-thiogalactopyranoside (IPTG) to produce RtxA/proRtxA; 3, clarified crude urea extract from induced cells; 4, Ni-NTA agarose column flowthrough; 5, Ni-NTA agarose column wash; 6, fraction of eluted 105-kDa RtxA/proRtxA; 7, phenyl-sepharose column flowthrough; 8, phenyl-sepharose column wash; 9, fraction of eluted RtxA/proRtxA; and St, molecular-mass standards. The samples were analyzed on 7.5% polyacrylamide gels and stained with Coomassie blue

RTX cytotoxins are produced by many gram-negative bacterial pathogens, including members of the genera of *Actinobacillus*, *Aggregatibacter*, *Bordetella*, *Escherichia*, *Mannheimia*, *Moraxella*, *Morganella*, *Pasteurella*, *Proteus* and *Vibrio*^17^. The *K. kingae* RTX locus encodes the RtxA cytotoxin and four other proteins whose functions were inferred from the known functions of homologous RTX proteins^11^. These include the toxin activation acyltransferase RtxC and three proteins (RtxB, RtxD and TolC) that form the type I secretion system (TISS). The TISS of *K. kingae* appears to be functional, since RtxA was identified as a secreted soluble protein in the extracellular medium of a *K. kingae* culture^18^. Based on homology with other RTX toxins^17^, several functional segments can be defined in the 956 residue RtxA polypeptide (Fig. 1a): (i) a hydrophobic pore-forming domain located between residues 140 to 410 that harbors four putative transmembrane α-helices; (ii) an acylated segment where the proRtxA protein is activated and converted to RtxA by the RtxC-catalyzed covalent post-translational acylation of two conserved lysine residues (K558 and K689); (iii) a typical calcium-binding RTX domain between residues 730 to 810 that harbors conserved nonapeptide repeats with the consensus sequence X-(L/I/F)-X-G-G-X-G-(N/D)-D, which form calcium-binding sites; and (iv) a carboxy-proximal secretion signal. RtxA binds and permeabilizes target cells and was observed to form cation-selective pores with an apparent diameter of 1.9 nm in artificial asolectin/n-decane membranes^19^.

Despite an increasing number of reports demonstrating that *K. kingae* is an important cause of various pediatric diseases, little is known about the molecular mechanisms by which RtxA interacts with host cells or the role of the toxin in the pathogenic process. To perform biological, functional and structural studies of RtxA, we overproduced the toxin in an *E. coli* expression system, developed a rapid procedure for its purification and elucidated the mechanism of RtxA activation. Using highly purified RtxA, we subsequently demonstrated that cholesterol facilitates the interaction of the toxin with artificial and cell membranes.

## Results

### Production and purification of recombinant RtxA and proRtxA

Biologically active RtxA is normally present in the cell culture supernatants of *K. kingae* isolates in low quantities, and its isolation and purification from bacterial cultures is a relatively complicated process. Therefore, we developed a procedure to overproduce and isolate a highly purified recombinant form of RtxA. The *rtxA* gene, encoding the inactive protoxin (proRtxA), and the *rtxC* gene, encoding a predicted fatty-acyltransferase that activates proRtxA, were PCR amplified from chromosomal DNA of the *K. kingae* septic arthritis isolate PYKK081 (ref^20^.) and cloned in the expression vector pT7–7 (ref^21^.). To express unmodified proRtxA, only the *rtxA* gene was inserted into pT7–7. Next, the *rtxA* gene was fused in frame at its 3′-terminus to a sequence encoding a double-hexahistidine purification tag. The RtxA and proRtxA proteins were expressed in *E. coli* BL21/pMM100 cells and purified from urea-solubilized inclusion bodies by a combination of affinity chromatography using Ni-NTA agarose and hydrophobic chromatography using phenyl-sepharose (Figs. 1b, c). The identities of the purified RtxA (Supplementary Table 1) and proRtxA (Supplementary Table 2) proteins were confirmed by peptide mass fingerprinting. This procedure yielded 10 to 15 mg of highly purified RtxA and proRtxA proteins per 1 L of bacterial culture.

### RtxA but not proRtxA is covalently acylated at lysine residues 558 and 689

**Fig. 2:**
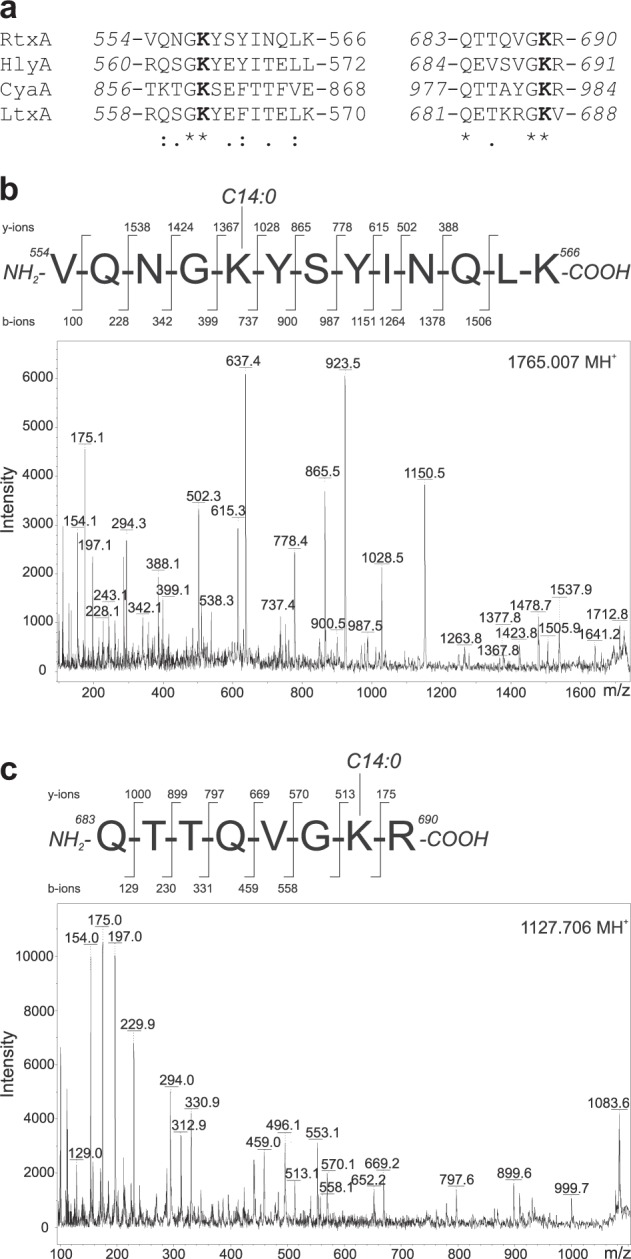
RtxA is acylated at lysine residues 558 and 689. **a** ClustalW sequence alignment of putative acylated sites of RtxA and corresponding sequences of related RTX toxins whose post-translational modification through amide-linked fatty acylation on the ε-amino group of two conserved internal lysine residues was previously demonstrated:^22, 23, 25, 26^ RtxA, *K. kingae* isolate PYKK081 cytotoxin; HlyA, *E. coli* α-hemolysin (UniProt code: Q8G9Z4); CyaA, *B. pertussis* adenylate cyclase toxin (UniProt code: P0DKX7); LtxA, *A. actinomycetemcomitans* leukotoxin (UniProt code: P16462). The highly conserved lysine residues (K558 and K689 in RtxA) that have been shown to be acylated in HlyA, CyaA and LtxA are shown in bold. Symbols: (*) identity; (:) strongly similar; (.) weakly similar. **b**, **c** The tryptic fragments with m/z signals 1099.675, 1127.706, 1143.699, 1153.723, 1765.007 and 1781.003, which were exclusively present in trypsin-digested RtxA (Supplementary Fig. 1), were further analyzed using a tandem MS/MS sequencing approach. **b** The MS/MS spectrum of the peptide 1765.007 contains b- and y-ions that correspond to the sequence 554-VQNGKYSYINQLK-566 with the ε-amino group of K558 modified by a myristoyl (C14:0) acyl chain. **c** The MS/MS spectrum of the peptide 1127.706 contains b- and y-ions that correspond to the sequence 683-QTTQVGKR-690 with a myristoyl (C14:0) acyl group attached to the ε-amino group of K689. The MS/MS spectra of the remaining unexpected peptides (1099.675, 1143.699, 1153.723 and 1781.003) are shown in Supplementary Fig. 2. The residue numbering corresponds to that of the full-length sequence of the RtxA variant from the *K. kingae* isolate PYKK081

Previous studies of other RTX toxins, namely the *B. pertussis* toxin CyaA^22–24^, the *E. coli* α-hemolysin HlyA^25,26^ and the *A. actinomycetemcomitans* leukotoxin LtxA^27^, demonstrated that they are post-translationally activated through amide-linked fatty acylation of the ε-amino groups of two conserved internal lysine residues. Sequence alignment analysis of these three RTX toxins with RtxA revealed lysine residues 558 and 689 of proRtxA to be putative acylation sites (Fig. 2a). To analyze the acylation of the purified proRtxA and RtxA proteins, they were separated by SDS-PAGE and digested in-gel by trypsin, after which the resulting peptides were separated by reversed-phase microcapillary high-performance liquid chromatography and analyzed by mass spectrometry (MS). The tryptic peptides with m/z signals that were exclusively present in the trypsin-digested RtxA sample (Supplementary Fig. 1) were further analyzed using a tandem MS/MS sequencing approach. As shown by the spectra in Fig. 2b and Supplementary Fig. 2a, MS/MS fragmentation of the unexpected peptides revealed two peptides with the sequence 554-VQNGKYSYINQLK-566 and an increase in mass of 210.198 and 226.193 Da on the K558 residue, indicating covalent modification by myristoyl (C14:0) and hydroxymyristoyl (C14:0-OH) chains, respectively. Four peptides had the sequence 683-QTTQVGKR-690 and exhibited mass increases of 182.167, 210.198, 226.193 and 236.214 Da on the K689 residue, suggesting the attachment of lauroyl (C12:0), myristoyl (C14:0), hydroxymyristoyl (C14:0-OH) and palmitoleyl (C16:1) chains, respectively (Fig. 2c and Supplementary Fig. 2b–d). The relative amounts of acylated peptides for the two acylation sites were semiquantitatively estimated from the relative intensities of selected ions in reconstructed ion current chromatograms. As summarized in Supplementary Table 3, myristoyl and hydroxymyristoyl chains were the most dominant acyl chains bound to the peptides, accounting for ~23 and ~89% of K558 and K689 acylation, respectively. Only a small proportion of the RtxA molecules (~10%) was covalently modified at the K689 residue by lauroyl and palmitoleyl chains (Supplementary Table 3).

### RtxA but not proRtxA exhibits cytotoxicity against human cells

**Fig. 3:**
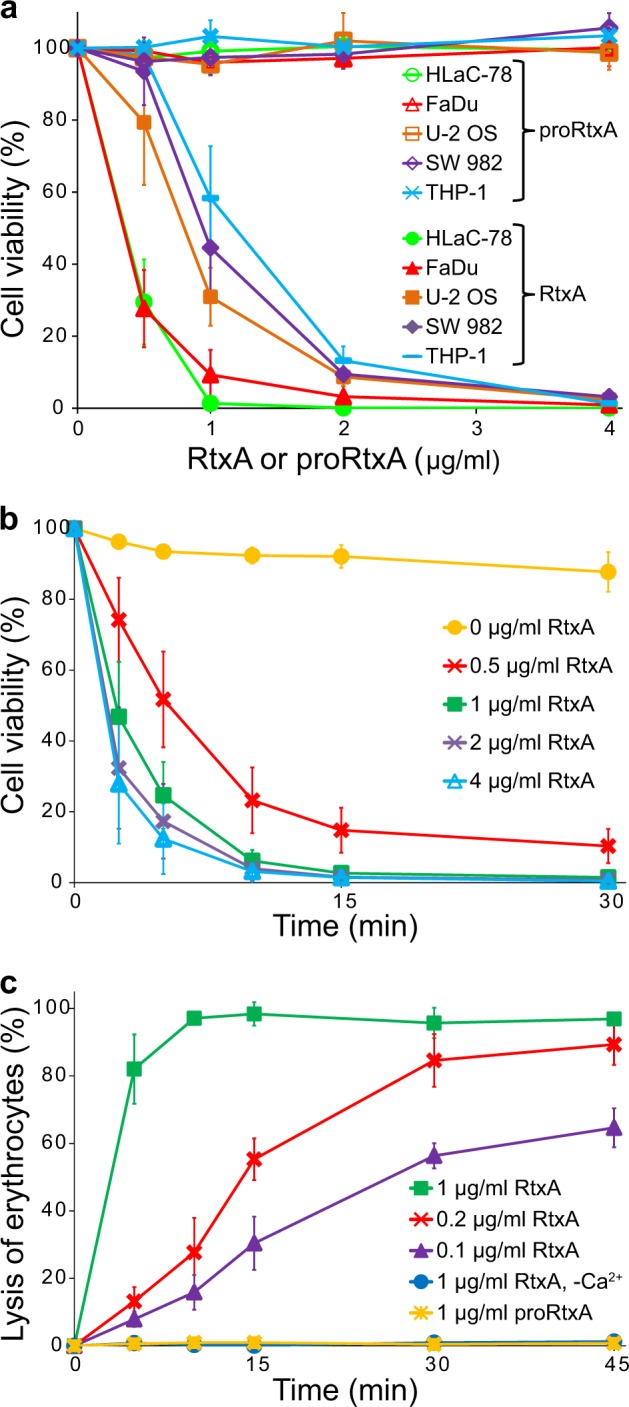
RtxA but not proRtxA is cytotoxic against various cells. **a** Different cell types (1 × 10^6^/ml) were incubated with increasing concentrations (0.5, 1, 2, and 4 µg/ml) of purified RtxA or proRtxA in the presence of 2 mM calcium ions for 1 h at 37 °C. **b** HLaC-78 cells (1 × 10^6^/ml) were incubated with the indicated concentrations of purified RtxA for different times at 37 °C. **a**, **b** Cell viability was determined by a cell viability staining assay using 1 µg/ml of Hoechst 33258 followed by flow cytometry. The viability of cells incubated without RtxA was reported as 100%. Each point represents the mean value ± SD of four independent experiments. **c** Erythrocytes (5 × 10^8^/ml) were incubated at 37 °C in the presence of increasing concentrations (0.1, 0.2, and 1 µg/ml) of purified RtxA or 1 µg/ml of proRtxA. Erythrocytes incubated with 1 µg/ml of RtxA in the absence of calcium ions were used as negative control. Cytolytic (hemolytic) activity was measured at different times as the amount of released hemoglobin by photometric determination (A_541nm_). Complete lysis of erythrocytes was reported as 100%. Each point represents the mean value ± SD of three independent determinations performed in duplicate

The cytotoxic activities of the acylated RtxA and the unacylated proRtxA were tested on different human cell types that may be attacked by the toxin during *K. kingae* infection, including laryngeal HLaC-78 squamous cells, hypopharyngeal FaDu epithelial cells, bone osteosarcoma U-2 OS epithelial cells, synovial SW 982 cells and monocytic THP-1 cells. The cells were exposed to different concentrations of the highly purified RtxA and proRtxA proteins, and the susceptibility of cells to RtxA/proRtxA-mediated killing was determined by a cell viability assay using the nucleic acid stain Hoechst 33258 followed by flow cytometry. As shown in Fig. 3a, the viability of all tested cells was reduced in a dose-dependent manner upon treatment with RtxA, while the viability of cells incubated with unacylated proRtxA remained unaffected over the 1-h testing period. The viability of the most susceptible cells, HLaC-78 and FaDu, was reduced by ~70 and ~90% upon incubation with 0.5 and 1 µg/ml of RtxA, respectively (Fig. 3a). The remaining three cell types, U-2 OS, SW 982 and THP-1, were approximately two-fold less susceptible to RtxA activity than the HLaC-78 and FaDu cells (Fig. 3a). The cells had to be incubated with RtxA in the presence of 2 mM calcium ions, since the viability of cells treated with the toxin in the absence of calcium ions remained unaffected (Supplementary Fig. 3). This requirement indicates that as with other RTX toxins, the cytotoxic activity of RtxA is strictly dependent on the presence of free calcium ions^17^. The results of a time course analysis of the cytotoxic effect of RtxA on HLaC-78 cells demonstrated that cell viability decreased rapidly, even at the lowest concentration of the toxin tested. As shown in Fig. 3b, the cell viability was reduced to 50% after HLaC-78 cells were incubated with 0.5 or 1 µg/ml of RtxA for 5 and 2.5 min, respectively. A complete loss of cell viability was observed after HLaC-78 cells were incubated with 1 µg/ml of RtxA for 15 min (Fig. 3b).

We further tested the cytotoxic effect of the recombinant RtxA toxin on sheep erythrocytes, which are commonly used as a simple model to analyze the hemolytic (cytolytic) activity of some RTX toxins^28,29^. Sheep erythrocytes were incubated with different concentrations of purified RtxA, and the hemolytic activity was measured as the amount of hemoglobin released over time by photometric determination. As shown in Fig. 3c, ~50% of erythrocytes lysed after exposure to 0.2 µg/ml of RtxA for 15 min, and the complete lysis of erythrocytes was observed when the cells were incubated with 1 µg/ml of the toxin for 10 min. No lysis over the 45-min testing period was observed when erythrocytes were incubated with 1 µg/ml of RtxA in the absence of calcium ions or when incubated with 1 µg/ml of proRtxA (Fig. 3c).

These results demonstrate that the purified recombinant RtxA toxin exerts calcium-dependent cytotoxicity toward various cell types and that this cytotoxicity is dependent on acylation of the K558 and/or K689 residues.

### RtxA loses its cytotoxic activity under native conditions

**Fig. 4:**
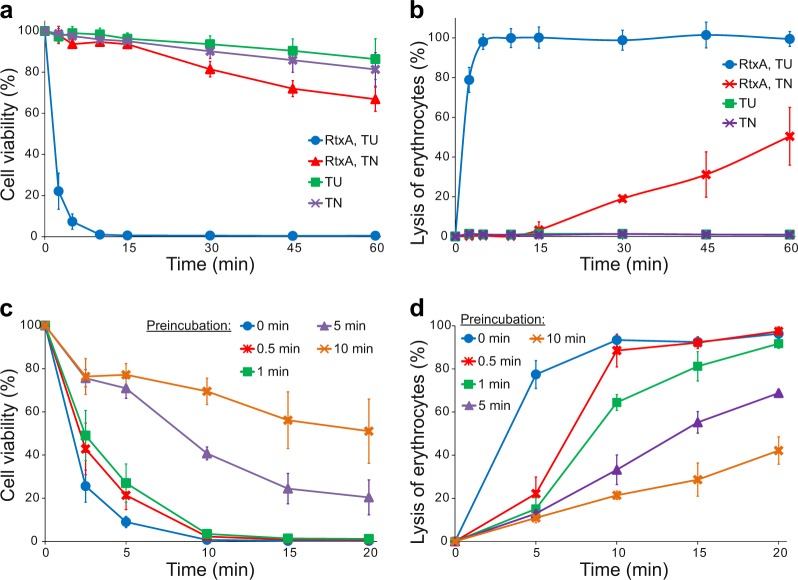
RtxA loses its cytotoxic activity under native conditions. **a**, **b** RtxA purified under denaturing conditions (50 mM Tri-HCl and 8 M urea; RtxA, TU) and RtxA purified under native conditions (50 mM Tris-HCl and 150 mM NaCl; RtxA, TN) were diluted 100-fold into HLaC-78 cells (1 × 10^6^/ml) **(a)** and erythrocytes (5 × 10^8^/ml) **(b)** to a final concentration of 2 µg/ml. TU and TN buffers without RtxA were used as negative controls. Cells were incubated for different times at 37 °C and analyzed as described in the Fig. 3 legend. Each point represents the mean value ± SD of four independent experiments. **c**, **d** RtxA purified under denaturing conditions was diluted 100-fold with TN buffer to concentrations of 2 and 0.5 µg/ml, respectively. The toxin samples were preincubated for different times (0, 0.5, 1, 5, and 10 min) at 37 °C and were subsequently added to pelleted HLaC-78 cells (1 × 10^6^/ml; from 2 µg/ml RtxA samples) **(c)** and erythrocytes (5 × 10^8^/ml; from 0.5 µg/ml RtxA samples) **(d)**. The cells were incubated for different times at 37 °C and analyzed as described in the Fig. 3 legend. Each point represents the mean value ± SD of four independent experiments **(c)** or three independent determinations performed in duplicate **(d)**

All the experiments described above were performed with RtxA purified from inclusion bodies under denaturing conditions in 8 M urea, since we observed that the toxin had low cytotoxic activity toward target cells when it was purified from bacterial cytosol under native conditions (Figs. 4a, b). Moreover, RtxA that was purified in 8 M urea had to be diluted (100×) into native buffer immediately before being added to cells, as a longer preincubation of the toxin in native buffer resulted in the rapid loss of its cytotoxic activity. As shown in Fig. 4c, HLaC-78 cell viability was reduced by ~90% after incubating for 5 min with 2 µg/ml of RtxA that was directly 100-fold diluted from the RtxA stock in 8 M urea. In contrast, when the toxin was first prediluted in native buffer and preincubated for 1, 5 or 10 min before being added to HLaC-78 cells, cell viability was only reduced by ~70, 30, and 20%, respectively. Similarly, erythrocytes incubated for 10 min with 0.5 µg/ml of RtxA that was directly diluted from the RtxA stock in 8 M urea were lysed to 90%, while erythrocytes treated with RtxA that was preincubated in native buffer were lysed less efficiently (Fig. 4d).

### Recombinant RtxA forms pores in artificial membranes with substantially higher overall membrane activity than proRtxA

**Fig. 5:**
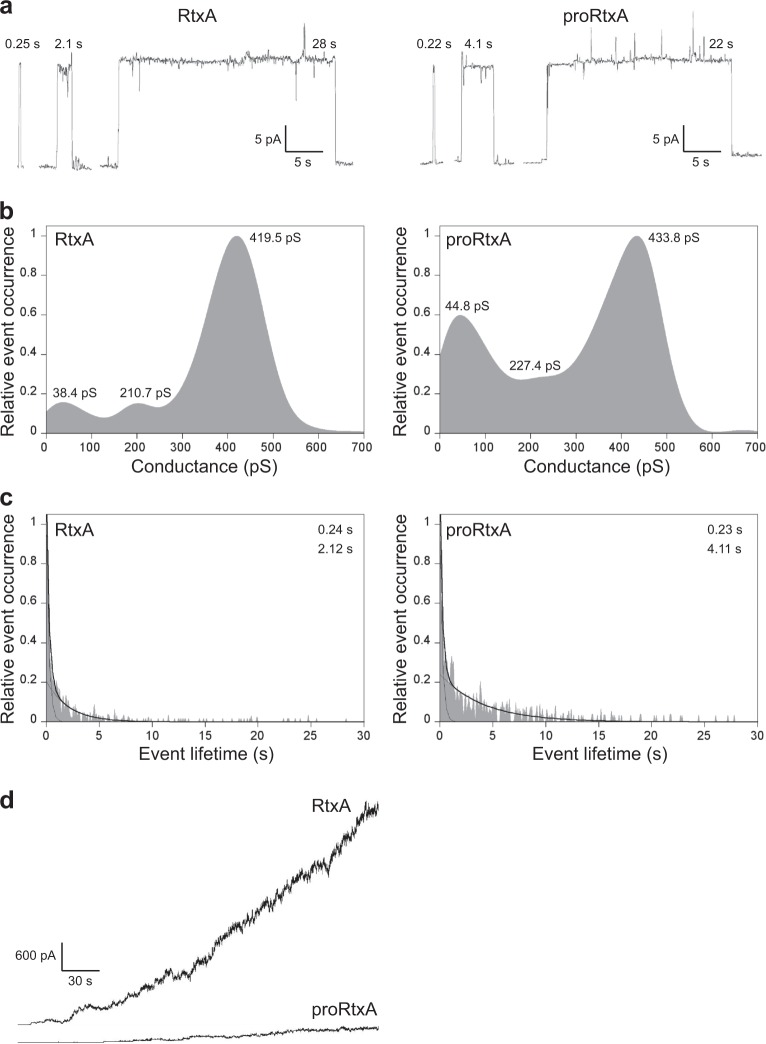
Conductances, lifetimes and overall membrane activities of RtxA and proRtxA on planar lipid membranes. **a** Representative single-pore recordings of asolectin/decane:butanol (9:1) membranes in the presence of 1 nM purified recombinant RtxA and proRtxA. The aqueous phase contained 150 mM KCl, 10 mM Tris-HCl (pH 7.4), 2 mM CaCl_2_; the applied voltage was 50 mV; the temperature was 25 °C and the recording was filtered at 10 Hz. The numbers above the recordings represent lifetimes. **b** Kernel density estimation (KDE) of single-pore conductances of RtxA and proRtxA (>500 events) were calculated from single-pore recordings acquired on several different asolectin membranes under the same conditions as in (**a**). The results are summarized in Table 1, and the numbers in each KDE represent the most frequent values of three well-distinguishable single-pore conductance states. **c** For lifetime determination, approximately 400 individual pore openings were recorded on several different asolectin membranes with 0.25, 0.5, and 1 nM RtxA or proRtxA (all other parameters were the same as in (**a**)), and the KDE of dwell times was fitted with a double-exponential function. The error estimates of lifetimes were obtained by bootstrap analysis. The results are summarized in Table 1, and the numbers in each graph represent the most frequent values of two well-distinguishable lifetimes. **d** Overall membrane activity of the purified RtxA and proRtxA proteins on asolectin/decane:butanol (9:1) membranes using the same conditions as in (a)

To characterize the membrane pores formed by the purified recombinant RtxA and proRtxA proteins at the molecular level, we analyzed the single-pore conductance and lifetime of pores formed upon incubation of artificial planar lipid bilayers made of 3% asolectin with RtxA and proRtxA. As shown in Fig. 5 and summarized in Table 1, RtxA generated pores (Fig. 5a) with three well-distinguishable conductance states, with the most frequently observed values being 38.4, 210.7, and 419.5 pS (Fig. 5b and Table 1). The majority of pores formed by RtxA exhibited short lifetimes, with 0.24 s being the most frequently observed value, while the remaining toxin-generated pores exhibited longer lifetimes, with 2.12 s being the most frequently observed value (Fig. 5c and Table 1). A minor proportion of RtxA pores were opened for tens of seconds (Fig. 5a). The proRtxA protoxin formed pores with similar single-pore conductance states (44.8, 227.4, and 433.8 pS) and a most frequent shorter lifetime (0.23 s) as the RtxA toxin (Fig. 5a–c and Table 1). The most frequent longer lifetime of proRtxA was 4.11 s, two times higher than that of RtxA (Fig. 5c and Table 1). However, the acylated RtxA and unacylated proRtxA importantly differed in their overall membrane activity (i.e., the combined contributions of the number of pore-forming molecules in the membrane, conductance and lifetime of single pores, and the frequency of pore formation), which was 8.3 pA/s for RtxA and only 1.1 pA/s for proRtxA (Fig. 5d and Table 1). These results indicate that proRtxA was impaired in its ability to correctly insert into the lipid bilayer and/or in its propensity to form oligomeric pores with the same frequency as the acylated toxin.

**Table 1:** Activities of RtxA and proRtxA on planar lipid membranes

Protein^a^	Most frequent values of single-pore conductance states with frequency of occurrence^a^			Most frequent values of lifetimes with frequency of occurrence^b^		Overall membrane activity (pA/s)^c^
	G1 (pS)	G2 (pS)	G3 (pS)	τ1 (s)	τ2 (s)	
RtxA	38.4 ± 57.4 (10%)	210.7 ± 66.3 (10%)	419.5 ± 74.3 (80%)	0.24 ± 0.06 (79%)	2.12 ± 0.48 (21%)	8.3
proRtxA	44.8 ± 72.7 (28%)	227.4 ± 99.2 (17%)	433.8 ± 83.3 (55%)	0.23 ± 0.16 (78%)	4.11 ± 0.11 (22%)	1.1

^a^Single-pore conductance states of RtxA and proRtxA (1 nM) were determined in 150 mM KCl, 10 mM Tris-HCl (pH 7.4) and 2 mM CaCl_2_ at 25 °C with an applied voltage of 50 mV. The most frequent values of three well-distinguishable single-pore conductance states (G1, G2 and G3) for each protein ± S.D. (half-width at half maximum) are shown. The percentage of the frequency of occurrence for each single-pore conductance state is shown in parentheses

^b^For lifetime determination, the kernel density estimation of dwell times (of ~400 individual pore openings) was fitted with a double-exponential function. The error estimates of lifetimes were obtained by bootstrap analysis. The most frequent values of two well-distinguishable lifetimes (τ1 and τ2) for each protein ± S.D. are shown. The percentage of the frequency of occurrence for each lifetime is given in parentheses

^c^The overall membrane activity of RtxA and proRtxA was determined in asolectin membranes under the same conditions as in (^a^) for 5 min and reflects the number of pore-forming molecules in the membrane, conductance (size) and lifetime of single pores, and the frequency of the formation of pores by the proteins

### Cholesterol is important for the binding and cytotoxic activity of RtxA

**Fig. 6:**
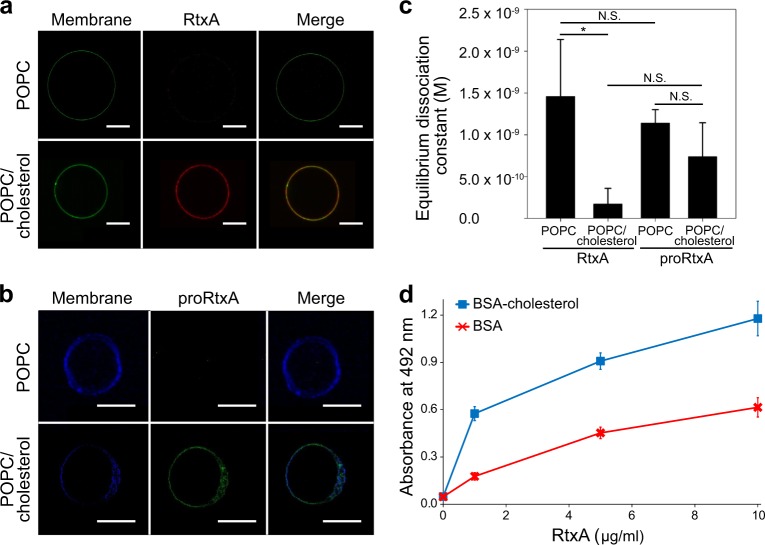
Cholesterol increases membrane binding of RtxA and proRtxA. **a** RtxA labeled with AF555 (red) was incubated with GUVs composed of either 100% POPC or 75% POPC and 25% cholesterol, labeled with NBD-PE (green) for 30 min. **b** proRtxA labeled with Dy495 (green) was incubated with GUVs composed of either 100% POPC or 75% POPC and 25% cholesterol, labeled with Marina Blue (blue) for 30 min. **c** RtxA and proRtxA binding to POPC and POPC/cholesterol membranes was analyzed by SPR. The equilibrium dissociation constant (K_D_) was measured for RtxA and proRtxA binding to 100% POPC or 75% POPC and 25% cholesterol membranes by determining the association and dissociation rate constants of each interaction (Supplementary Table 4). *p < 0.05; N.S., not significant. **d** Wells of a 96-well plate were coated with 10 μg/ml of cholesterol-BSA or free BSA, which were subsequently incubated with different concentrations of purified RtxA that was detected with the mAb 9D4. Each point represents the mean value ± SD of three independent determinations performed in duplicate

The presence of cholesterol in the cell membrane was previously observed to be important for the cytotoxic activities of some RTX toxins, two of which, LtxA and HlyA, have been shown to specifically bind cholesterol^30–32^. To examine if cholesterol has a role in the membrane binding of RtxA and proRtxA, we visualized the affinities of both proteins to 1-palmitoyl-2-oleoyl-*sn*-glycero-3-phosphocholine (POPC) and POPC/cholesterol (75/25) membranes using a fluorescent giant unilamellar vesicle (GUV) assay^30^. RtxA was labeled with AF555 (red) and GUVs were labeled with NBD-PE (green). As shown in Fig. 6a, little RtxA was observed to be associated with the 100% POPC membrane, but the toxin bound readily to GUVs composed of 75% POPC and 25% cholesterol. The experiment was repeated for proRtxA, where proRtxA was labeled with Dy495 (green) and GUVs were labeled with Marina Blue (blue). As with RtxA, lower binding of proRtxA was observed to 100% POPC GUVs than to cholesterol-containing GUVs (Fig. 6b).

The affinities of RtxA and proRtxA for membranes with and without cholesterol were quantified using surface plasmon resonance (SPR). As shown in Fig. 6c and summarized in Supplementary Table 4, the equilibrium dissociation constant (K_D_) of RtxA for POPC membranes was approximately 1.5 × 10^–9^ M, while that of proRtxA was slightly lower, although this difference was not significant. Consistent with other RTX toxins, including LtxA^30^, the K_D_ of RtxA for cholesterol-containing membranes was lower than its K_D_ for POPC membranes, indicating a stronger affinity for the POPC/cholesterol membrane (Fig. 6c and Supplementary Table 4). Similarly, the K_D_ of proRtxA for POPC/cholesterol membranes was lower than its K_D_ for POPC membranes, indicating a stronger affinity for the POPC/cholesterol membrane (Fig. 6c and Supplementary Table 4).

As shown in Supplementary Table 4, the association rates (k_a_) for each sample were similar. The dissociation rates (k_d_) of each toxin for each type of membrane were also similar, with the exception of RtxA binding to POPC/cholesterol, which was one order of magnitude slower than the other dissociation rates. This slower dissociation rate drives the increase in the overall affinity (K_D_) of this toxin for cholesterol-containing membranes relative to POPC membranes.

To analyze whether RtxA is able to interact with cholesterol in the absence of lipid membranes, the wells of a 96-well plate were coated with cholesterol conjugated to BSA or free BSA, with the latter used as a negative control. The coated wells were incubated with different concentrations of purified RtxA that was subsequently detected by the antibody 9D4, which recognizes the RTX domain of RtxA^18^. As shown in Fig. 6d, the RtxA toxin exhibited a 2–3-fold greater binding capacity to cholesterol-BSA than to free BSA at all tested concentrations.

**Fig. 7:**
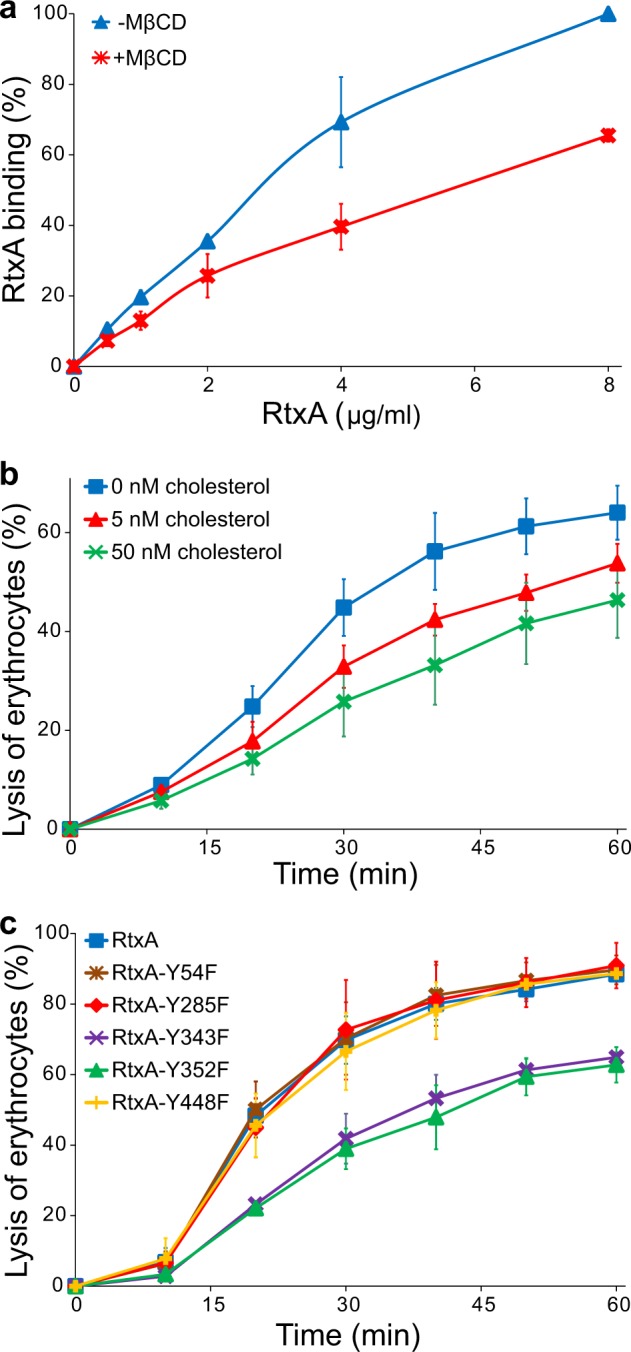
Cholesterol is important for the binding and cytotoxic activity of RtxA toward erythrocytes. **a** Erythrocytes (5 × 10^8^/ml) were pretreated with 1 mM MβCD at 37 °C for 30 min, diluted to 1 × 10^7^/ml and placed on ice before RtxA labeled with Dy495 was added at the indicated concentrations. The cells were incubated with the toxin for 15 min at 4 °C, and the amount of fluorescently labeled RtxA bound was determined by flow cytometry. The results are expressed as the relative binding of RtxA according to the following formula: relative binding = (sample binding)/(maximum binding) × 100. The data shown are the mean values ± SD of four independent experiments. **b** Purified RtxA (1 µg/ml) was preincubated for 10 min at room temperature in the presence (5 and 50 nM) or absence of cholesterol and then was added to erythrocytes (5 × 10^8^/ml). The cells were incubated for different times at 37 °C and analyzed as described in the Fig. 3 legend. Each point represents the mean value ± SD of three independent determinations performed in duplicate. **c** Substitutions of the tyrosine residues Y343 and Y352 by phenylalanine residues in the putative CARC/CRAC motifs reduce the cytolytic activity of RtxA. Erythrocytes (5 × 10^8^/ml) were incubated with 100 ng/ml of intact RtxA or its mutant variants for different times at 37 °C and analyzed as described in the Fig. 3 legend. The data shown are the mean values ± SD of three independent experiments

We further examined the role played by cholesterol in the membrane binding and cytotoxic activity of RtxA toward erythrocytes. First, erythrocytes were preincubated in the absence or in the presence of 1 mM methyl-β-cyclodextrin (MβCD), which is commonly used to deplete membrane cholesterol^33,34^. Next, the cells were incubated with different concentrations of fluorescently labeled RtxA, and binding of the toxin to erythrocytes was analyzed by flow cytometry. As shown in Fig. 7a, RtxA bound with an approximately 30 to 40% lower efficiency to MβCD-treated cells compared with untreated cells, indicating that membrane cholesterol facilitates binding of the toxin to the cell surface.

To analyze whether free cholesterol decreases the cytotoxic activity of RtxA, the toxin was preincubated with 5 or 50 nM of free cholesterol and was subsequently incubated in the presence of free cholesterol with erythrocytes for different periods of time. As shown in Fig. 7b, the exposure of RtxA to free cholesterol significantly reduced its hemolytic activity over the 1-h testing period.

Taken together, these results demonstrate that the interaction of RtxA with the target membrane is enhanced by membrane cholesterol, which directly interacts with the toxin.

### Phenylalanine substitutions of the Y343 and Y352 residues in the putative cholesterol binding motifs reduce the cytolytic activity of RtxA

A primary sequence analysis of RtxA revealed five potential cholesterol binding sites located within or adjacent to the predicted pore-forming domain (residues 140 to 410) of the toxin (Fig. 1a and Supplementary Table 5). Two of these sites are so-called recognition/interaction amino acid consensus (CRAC) motifs, consisting of the pattern L/V-(X)(1–5)-Y-(X)(1–5)-R/K (where (X)(1–5) represents between one and five residues of any amino acid)^35^, and three are CARC motifs, which are inverted CRACs with the consensus sequence R/K-(X)(1–5)-Y/F-(X)(1–5)-L/V^36^. The presence of these motifs suggests that RtxA may directly interact with host cells through these predicted CRAC/CARC motifs and membrane cholesterol, as has been demonstrated for the RTX toxin LtxA^30,31^. To test which of the predicted CRAC/CARC motifs in RtxA mediate membrane cholesterol binding, we replaced the key tyrosine residues of these motifs (Y54, Y285, Y343, Y352, and Y448) with phenylalanine residues. As shown in Fig. 7c, the Y54F, Y285F, and Y448F substitutions in the putative CRAC/CARC motifs did not significantly affect the lytic activity of the RtxA mutants toward erythrocytes. In contrast, the Y343F and Y352F substitutions in the CARC_340–348_ and CRAC_349–354_ motifs, respectively, reduced the lytic activity of the mutant RtxA proteins to ~50% that of wild-type RtxA after a 20 min incubation of the toxins with erythrocytes (the half time of cell lysis with RtxA; Fig. 7c). Thus, the tandem CARC-CRAC motifs located between residues 340–354 of RtxA may be involved in the interaction between the toxin and membrane cholesterol.

## Discussion

The *K. kingae* RtxA toxin belongs to a broad family of the secreted RTX cytotoxins, which are gaining attention as important virulence factors of various gram-negative bacterial pathogens^17^. While some RTX toxins, such as the *E. coli* α-hemolysin HlyA and the *B. pertussis* adenylate cyclase toxin CyaA, have been intensively studied over the last decades^17,37–39^, the cytotoxin RtxA is one of the least characterized members of the RTX toxin family. To perform biological, biochemical, biophysical and structural studies of RtxA, we developed a simple procedure for the overproduction and purification of milligram amounts of biologically active recombinant RtxA. Using different approaches, we have demonstrated that the cytotoxic activity of RtxA depends on post-translational acylation of its lysine residues 558 and/or 689 and that cholesterol facilitates the interaction of the toxin with artificial and cell membranes.

Although it is generally accepted that the RTX toxins are post-translationally acylated on the ε-amino groups of two conserved lysine residues, the type of acyl chains has only been experimentally identified for three RTX toxins, namely the *B. pertussis* CyaA^22–24^, the *E. coli* α-hemolysin HlyA^25,26^ and the *A. actinomycetemcomitans* leukotoxin LtxA^27^. Analysis of the predicted acylated segment of RtxA and its alignment with the corresponding regions of CyaA, HlyA and LtxA revealed that the RtxA protein contains two conserved lysine residues, K558 and K689 (Fig. 2a), that could be post-translationally modified. This modification was experimentally confirmed by MS analyses of the RtxA protein coexpressed with a putative acyltransferase (RtxC), which revealed that the K558 residue was modified by C14:0 and C14:0-OH fatty acyl groups (Fig. 2b and Supplementary Fig. 2a), whereas the K689 residue was modified by several fatty acyl groups, including C12:0, C14:0, C14:0-OH and C16:1 (Fig. 2c and Supplementary Fig. 2b-d). As demonstrated recently, C12:0, C14:0 and C16:1 are the major fatty acids present in *K. kingae* ATCC 23330^T^ and *K. negevensis* Sch538^T^ (ref^40^.). The saturated C14:0 acyl group was observed to be the most dominant acyl form, accounting for ~18 and ~71% of K558 and K689 acylation of RtxA, respectively (Supplementary Table 3). This result was similar to that described for HlyA, where the C14:0 acyl group was observed to constitute 68% of the acyl chains attached to the K564 and K690 residues^26^. The second most abundant acyl group linked to RtxA, a monohydroxylated form of C14:0 (Supplementary Table 3), was previously identified in LtxA^27^. Only a small proportion of the RtxA molecules (~8%) were covalently modified at the K689 residue by the monounsaturated C16:1 fatty acyl chain (Supplementary Table 3), which together with the saturated C16:0 acyl group are two major acyl modifications of the K860 and K983 residues of recombinant CyaA^23,24^. In contrast to the results of Lim et al. for HlyA^26^, but in agreement with those of Fong et al. for LtxA^27^, we were unable to identify odd-carbon fatty acyl groups, such as C15:0 and C17:0, attached to the RtxA molecule.

We demonstrated that similar to RtxA secreted by *K. kingae*^11^, the highly purified recombinant RtxA is a potent cytotoxin capable of rapid and efficient killing of various human cell types (Figs. 3a, b). Moreover, RtxA was able to efficiently lyse sheep erythrocytes (Fig. 3c) that are commonly used as a model to test the cytolytic activity of some other RTX toxins^28,41^. In contrast, the viability of cells incubated with unacylated proRtxA remained unaffected (Fig. 3a), and the protoxin was unable to lyse erythrocytes (Fig. 3c). Our data are in agreement with many previous studies showing that the acylation of RTX toxins is crucial for their cytotoxic activities^25,42,43^. Although the exact molecular mechanism by which the acylation contributes to the toxicity of the RTX toxins is unknown, the acyl chains have been demonstrated to have a structural role in supporting the folding of CyaA into biologically active states^44^, are responsible for the irreversible binding of HlyA to target membranes^45^, and/or promote protein-protein interactions between HlyA monomers to enable toxin oligomerization in the membrane during pore formation^46^. Therefore, we hypothesize that the acyl chains of RtxA may have a direct impact on these steps, which are also believed to be crucial for the cytotoxic activity of RtxA. In agreement with the inability of proRtxA to reduce cell viability (Fig. 3a) or to lyse erythrocytes (Fig. 3c), the unacylated protein displayed a substantially reduced overall membrane activity in planar lipid bilayers compared to RtxA (Fig. 5d). The unacylated protein was most likely impaired in its ability to bind/insert into the membrane and/or in its propensity to form oligomeric pores, as discussed above, since once inserted to the membrane, proRtxA formed pores with similar single-pore conductance states and lifetimes as RtxA (Fig. 5 and Table 1). This result is in agreement with previous studies showing that despite highly decreased overall membrane activity, the unacylated proHlyA and proCyaA proteins formed pores with similar properties as the acylated toxins^42,47,48^. The inefficient binding of proHlyA to the lipid bilayers^47^ and the highly reduced propensity of proCyaA to form membrane pores^42,48^ was suggested to be responsible for the reduced membrane activity.

We observed that when expressed as recombinant protein in *E. coli* and purified from urea-solubilized inclusion bodies under denaturing conditions in 8 M urea, RtxA was much more active against target cells than when the toxin was purified according the same scheme from the bacterial cytosol under native conditions (Figs. 4a, b). Moreover, to attain maximal biological activity, denatured and stabilized RtxA stored in 8 M urea had to be diluted into urea-free buffer just prior to being added to cells, since preincubation of RtxA in the native buffer resulted in a rapid reduction in its cytotoxic activity (Figs. 4c, d). This result indicates that RtxA has a tendency to self-aggregate and lose cytotoxic activity in solutions that do not contain denaturing concentrations of chaotropic agents. A similar loss of biological activity was described for other members of the RTX toxin family, which also tend to form biologically inactive aggregates in buffers that do not contain high concentrations of chaotropic agents^44,49–53^. Self-aggregation and inactivation of the RTX toxins has been proposed to result from the exposure and interaction of hydrophobic segments that are predominantly present in the membrane-binding pore-forming domains of the toxin molecules^44,51,53^. Therefore, RtxA and other RTX toxins have to be diluted into urea-free buffers just prior to addition to target cells to minimize the time for formation of toxin self-aggregates. In the presence of target cells, the hydrophobic regions of the toxins can directly interact with and insert into cell membranes, minimizing the rapid inactivation of the toxin molecules and resulting in highly specific cytotoxic activity of the RTX toxins^44,49–53^.

The RTX toxins HlyA and LtxA have been shown to specifically interact with lipid monolayers, liposomes and/or target cells through membrane cholesterol^30–32^, which is an essential structural component of all animal cell membranes. In this study, we used several different approaches to demonstrate that RtxA also has an affinity for cholesterol. We clearly visualized binding of RtxA and proRtxA to GUVs composed of 75% POPC and 25% cholesterol, whereas only weak binding of both proteins to GUVs made of 100% POPC was observed (Figs. 6a, b). In agreement with this result, stronger affinities of RtxA and proRtxA for the cholesterol-containing POPC membranes than for pure POPC membranes were observed via SPR (Fig. 6c and Supplementary Table 4). No significant difference in binding of either the acylated RtxA or the unacylated proRtxA to the cholesterol-containing POPC membranes was observed (Fig. 6c), indicating that the acyl chains of RtxA are not involved in its ability to bind cholesterol. Using an ELISA assay, we demonstrated that the toxin exhibits a 2–3-fold greater ability to bind cholesterol-BSA than free BSA (Fig. 6d), indicating that RtxA can bind cholesterol independently of the presence of other biological membrane components. In line with this result, preincubation of RtxA with free cholesterol significantly reduced its cytolytic activity toward erythrocytes (Fig. 7b). In addition, decreasing the cholesterol content of erythrocyte membranes by extraction with MβCD resulted in a 30–40% reduction in RtxA binding to MβCD-treated cells (Fig. 7a), indicating that membrane cholesterol facilitates binding of RtxA to the cell surface.

Many proteins that interact with membrane cholesterol have been shown to possess specific cholesterol binding motifs, of which the linear cholesterol binding motif CRAC and its reversed form CARC are well-known and widespread among cholesterol-binding proteins of diverse functions^54–56^. Two CRAC motifs were identified in the RTX leukotoxin LtxA, but only the CRAC_333–339_ motif, highly conserved among RTX toxins, in the putative membrane-interacting domain (residues 1–420) of LtxA was shown to be required for its toxicity^30^. Seven CRAC and thirteen CARC motifs were also predicted within the RTX hemolysin HlyA sequence, but their involvement in the interaction of HlyA with membrane cholesterol has not been experimentally demonstrated^32^. In this study, we identified five putative CRAC and CARC motifs located within or adjacent to the predicted pore-forming domain of RtxA (residues 140 to 410) (Fig. 1a and Supplementary Table 5). Assays using a panel of RtxA CRAC/CARC mutants demonstrated that the CARC_340–348_ and CRAC_349–354_ motifs located between residues 340–354 are required for the full cytolytic activity of RtxA (Fig. 7c). Since both of these motifs are adjacent to each other in the predicted membrane-interacting and pore-forming domain of RtxA, it is tempting to speculate that this CARC-CRAC tandem may be involved in the interaction of the toxin with membrane cholesterol. Indeed, the presence of both CARC and CRAC within the same transmembrane segment was shown to allow for its interaction with two cholesterol molecules^55^.

In summary, we developed an *E. coli* expression system that enables the production of milligram amounts of highly purified and biologically active RtxA. We expect that this system will greatly facilitate structural and functional studies of this key virulence factor, eliminating the need for time-consuming genetic manipulations and RtxA production in *K. kingae*. The production of recombinant RtxA allowed us to demonstrate that the cytotoxic activity of this protein depends on post-translational acylation of the K558 and/or K689 residues and on its ability to bind to membrane cholesterol.

## Materials and methods

### Bacterial strains

The *K. kingae* septic arthritis isolate PYKK081 (ref^20^.) was grown on Columbia agar with 5% sheep blood under a 5% CO_2_ atmosphere at 37 °C for 24 h. The *E. coli* strain XL1-Blue (Stratagene, La Jolla, CA) was used throughout this study for DNA manipulations and was grown in Luria-Bertani medium at 37 °C. The *E. coli* strain BL21 (Novagen, Madison, WI) carrying the plasmid pMM100 (encoding LacI and tetracycline resistance) was used to express the RtxA proteins.

### Human cell lines

Hypopharyngeal FaDu epithelial cells (ATCC HTB-43), monocytic THP-1 cells (ATCC TIB-202), bone osteosarcoma U-2 OS epithelial cells (ATCC HTB-96) and synovial SW 982 cells (ATCC HTB-93) were obtained from the American Type Culture Collection (ATCC, Manassas, VA). Laryngeal HLaC-78 squamous cells were established by Zenner at al^57^. FaDu, HLaC-78 and THP-1 were cultured in RPMI 1640 (Sigma-Aldrich, St. Louis, MO) supplemented with 10% fetal calf serum (FCS) (GIBCO Invitrogen, Grand Island, NY) and an antibiotic-antimycotic solution (0.1 mg/ml streptomycin, 1000 U/ml penicillin and 0.25 mg/ml amphotericin; Sigma-Aldrich). U-2 OS and SW 982 cells were grown in Dulbecco’s Modified Eagle’s medium (DMEM, Sigma-Aldrich) supplemented with 10% FCS and antibiotic antimycotic solution.

### Plasmid construction

To construct a plasmid to overproduce proRtxA, the *rtxA* gene was amplified from *K. kingae* isolate PYKK081 (ref^20^.) genomic DNA and was cloned into the vector pT7–7 (ref^21^.), yielding pT7*rtxA*. To express RtxC-activated RtxA, the plasmid pT7*rtxC*-*rtxA* was constructed by amplifying the *rtxC* gene from PYKK081 genomic DNA and cloning it into pT7*rtxA*. Both the *rtxA* and *rtxC* genes were placed under the control of the strong transcriptional and translational initiation signals of gene 10 from bacteriophage T7 and of the lactose promoter-operator region. A sequence encoding a double-hexahistidine tag^58^ was fused in frame to the 3’-end of the RtxA-encoding gene. Oligonucleotide-directed PCR mutagenesis was performed to construct pT7*rtxC*-*rtxA*-derived plasmids for the expression of RtxA mutant variants.

### Protein production, purification, and labeling

The recombinant proRtxA, RtxA and RtxA mutant variants were produced in *E. coli* BL21/pMM100 cells and purified by a combination of affinity and hydrophobic chromatography. Dy495-NHS ester was used to label the proRtxA and RtxA proteins. The details of this procedure are provided in the Supplementary Information.

### MS analysis

The purified proRtxA and RtxA proteins were separated by SDS-PAGE, digested in-gel with trypsin, and the resulting peptides were analyzed in parallel via liquid chromatography-mass spectrometry (LC-MS, ESI-qTOF) or off-line peptide separation followed by matrix-assisted laser desorption/ionization time-of-flight MS analysis. All details for these procedures are provided in the Supplementary Information.

### Planar lipid bilayers

Measurements on planar lipid bilayers^59^ were performed in Teflon cells separated by a diaphragm with a circular hole bearing the membrane, as described in detail in the Supplementary Information.

### Cytotoxicity assay

Cultured cells were harvested, washed once in a HEPES-buffered salt solution (HBSS buffer; 10 mM HEPES (pH 7.4), 140 mM NaCl, and 5 mM KCl) supplemented with 2 mM CaCl_2_ and 2 mM MgCl_2_ (HBSS-Ca/Mg buffer) and diluted with the same buffer to 1 × 10^6^ cells/ml. For experiments in the absence of calcium ions, the cells were prepared in HBSS buffer supplemented with 2 mM EDTA (HBSS-EDTA buffer). The cells were subsequently incubated with the indicated concentrations of purified proRtxA or RtxA for specified times at 37 °C. Cell viability was determined by a cell viability staining assay using 1 µg/ml of Hoechst 33258 followed by flow cytometry on a FACS LSR II instrument (BD Biosciences, San Jose, CA)^60^. Data were analyzed using the FlowJo software (Tree Star, Ashland, OR), and appropriate gatings were used to exclude cell aggregates and dying/dead cells (Hoechst 33258-positive staining). Cell viability in each sample was calculated according to the following formula: (count of viable cells in a sample with proRtxA or RtxA/count of viable cells in a sample without proRtxA or RtxA) × 100.

### Hemoglobin release assay

Sheep erythrocytes (LabMediaServis, Jaromer, Czech Republic) stored in Alsever’s Solution (Sigma-Aldrich) were repeatedly washed with TNC buffer (50 mM Tris-HCl (pH 7.4), 150 mM NaCl and 2 mM CaCl_2_). The washed erythrocytes (5 × 10^8^/ml) were then incubated with the indicated concentrations of proRtxA, RtxA or the RtxA mutant variants in 1 ml of TNC buffer, and hemolytic activity was measured over time by photometric determination (A_541_) of the hemoglobin release.

### GUV preparation

GUVs with compositions of POPC (99% POPC, 1% N-(7-nitrobenz-2-oxa-1,3-diazol-4-yl)−1,2-dihexadecanoyl-sn-glycero-3-phosphoethanolamine (NBD-PE) or Marina Blue) or POPC/cholesterol (74% POPC, 25% cholesterol, and 1% NBD-PE or Marina Blue) were created by mixing the required amounts of lipids dissolved in chloroform to a final lipid concentration of 4 mg/ml. The lipid/chloroform mixture was spin-coated onto a glass slide coated with indium tin oxide (SPI, West Chester, PA). Next, the slides were dried under vacuum for 30 min to remove any residual solvent. A polydimethylsiloxane spacer was used to separate two slides, creating a compartment that was filled with ultrapure water and sealed. Subsequently, an electric field was applied to the setup for 3 h at room temperature to form GUVs, which were used the same day they were prepared.

### Confocal microscopy

The proRtxA and RtxA proteins were labeled with Dy495-NHS ester (Dyomics, Jena, Germany) as described in the Supplementary Information or with Alexa Fluor (AF) 555 NHS Ester (Molecular Probes, Eugene, OR) according to the manufacturer’s instructions. GUVs were incubated with 30 ng of the fluorescently labeled proteins in ibiTreat µ-dishes (Ibidi, Martinsried, Germany) for 30 min. Imaging was conducted using a Nikon C2si + confocal microscope equipped with an LU-N4S laser unit and a 60 × oil objective (NA = 1.4). The images were processed using Nikon’s imaging software suite, Elements v.4.3.

### Liposome preparation for surface plasmon resonance (SPR) and SPR of proRtxA and RtxA

SPR was performed to quantify the affinities of RtxA and proRtxA for membranes with and without cholesterol as described in the Supplementary Information.

### Enzyme-linked immunosorbent assay (ELISA)

Cholesterol-captured ELISA was performed to analyze whether RtxA interacts with cholesterol in the absence of lipid membranes. The details of this assay are provided in the Supplementary Information.

### RtxA binding to cholesterol-depleted erythrocytes

Washed sheep erythrocytes (5 × 10^8^/ml) in TNC buffer were preincubated at 37 °C for 30 min in the absence or presence of 1 mM MβCD. Next, the cells were washed with TNC buffer, diluted to 1 × 10^7^/ml with TNC buffer supplemented with 75 mM sucrose (TNCS buffer), placed on ice and incubated with different concentrations of purified and Dy495-labeled RtxA for 15 min at 4 °C. Finally, the cells were washed with and resuspended in cold TNCS buffer, and the amounts of fluorescently labeled RtxA bound to the surface of erythrocytes were determined by flow cytometry.

### Statistical analysis

The results were expressed as the arithmetic means ± the standard deviation (SD) of the mean. Statistical analysis was performed by one-way ANOVA followed by Dunnett’s post-test using GraphPad Prism 6.0 (GraphPad Software, La Jolla, CA).

## Electronic supplementary material

Supporting Information
