# Supplementary material for: Cytotoxic activity of Kingella kingae RtxA toxin depends on post-translational acylation of lysine residues and cholesterol binding
Source: Emerg Microbes Infect. 2018 Nov 7;7:178. doi: 10.1038/s41426-018-0179-x (PMC6221878; doi:10.1038/s41426-018-0179-x)
Supplement: Supplementary file 1 — Supporting Information [file 41426_2018_179_MOESM1_ESM.docx]

*Supplementary Information*

**Cytotoxic activity of *Kingella kingae* RtxA toxin depends on post-translational acylation of lysine residues and cholesterol binding**

Adriana Osickova^1,2^, Nataliya Balashova^3^, Jiri Masin^1^, Miroslav Sulc^1,2^, Jana Roderova^1^, Tomas Wald^1,5^, Angela C. Brown^4^, Evan Koufos^4^, En Hyung Chang^4^, Alexander Giannakakis^3,6^, Edward T. Lally^3^ and Radim Osicka^1^

^1^Institute of Microbiology of the CAS, v.v.i., Prague, Czech Republic

^2^Faculty of Science, Charles University, Prague, Czech Republic

^3^Department of Pathology, School of Dental Medicine, University of Pennsylvania, Philadelphia, PA, USA

^4^Department of Chemical and Biomolecular Engineering, Lehigh University, Bethlehem, PA, USA

^5^Present address: Department of Orofacial Sciences and Program in Craniofacial Biology, University of California, San Francisco, CA, USA

^6^Present address: The Department of Cell and Molecular Biology at Karolinska Institutet, Stockholm, Sweden

Correspondence: Radim Osicka (osicka@biomed.cas.cz)

These authors contributed equally: Adriana Osickova, Nataliya Balashova, Radim Osicka

**Materials and methods**

**Protein production, purification and labeling**

The recombinant proRtxA, RtxA and RtxA mutant variants were produced in *E. coli* BL21/pMM100 cells transformed with the appropriate plasmids. Fifty-ml cultures were grown with shaking at 37 °C in MDO medium (yeast extract, 20 g/l; glycerol, 20 g/l; KH_2_PO_4_, 1 g/l; K_2_HPO_4_, 3 g/l; NH_4_Cl, 2 g/l; Na_2_SO_4_, 0.5 g/l; thiamine hydrochloride, 0.01 g/l) containing 150 μg/ml of ampicillin and 12.5 μg/ml of tetracycline. When cultures reached OD_600_ = 0.8, they were induced with 1 mM isopropyl β-D-1-thiogalactopyranoside (IPTG) and grown for additional 4 h. For protein purification, the cells were harvested by centrifugation, washed twice with 50 mM Tris-HCl (pH 7.4), 150 mM NaCl, 5 mM EDTA and disrupted by sonication at 4 °C. The homogenate was centrifuged at 20,000 x g for 30 min at 4 °C and the inclusion bodies collected in the pellet were washed with TN buffer (50 mM Tris-HCl (pH 7.4), 600 mM NaCl) containing 4 M urea and then solubilized with TN buffer containing 8 M urea and 5 mM imidazole. The urea extract was cleared at 20,000 x g for 30 min at 4 °C and loaded on an Ni-NTA agarose column (Qiagen, Germantown, MD) equilibrated with TN buffer containing 8 M urea and 80 mM imidazole. The column was washed with TN containing 8 M urea and 80 mM imidazole and RtxA was eluted with TN containing 8 M urea and 600 mM imidazole. The eluted fractions of RtxA were diluted 4-times in ice-cold 50 mM Tris-HCl, pH 8.0 containing 1 M NaCl and loaded on a Phenyl-Sepharose CL-4B (Sigma–Aldrich, St. Louis, MO) column equilibrated with the same buffer. Then, the column was washed with 50 mM Tris-HCl (pH 8.0) and RtxA was eluted with TU buffer (50 mM Tris-HCl (pH 8.0) and 8 M urea). To purify RtxA under native conditions, the homogenate after sonication of bacterial cells was cleared at 20,000 x g for 30 min at 4 °C and loaded on an Ni-NTA agarose column equilibrated with TN buffer and 80 mM imidazole. The column was washed with TN containing 80 mM imidazole and RtxA was eluted with TN containing 600 mM imidazole. Finally, imidazole was removed on a Sephadex G-25 (Pharmacia, Uppsala, Sweden) column equilibrated with TN buffer.

On-column labeling of proRtxA and RtxA was performed after the Ni-NTA agarose purification step. The protein sample was diluted 4-times in ice-cold 50 mM Tris-HCl, pH 8.0 containing 1 M NaCl and loaded on Phenyl-Sepharose beads. The Phenyl-Sepharose column was washed with 50 mM sodium bicarbonate (pH 8.3) and the beads were subsequently resuspended in the same buffer containing Dy495-NHS ester (Dyomics, Jena, Germany) in a concentration to reach a Dy495:protein molar ratio of ~6:1. Labeling was performed at 25 °C for 2 h, the column was washed with 50 mM Tris-HCl (pH 8.0) and labeled proteins were eluted in TU buffer.

**Mass spectrometry (MS) analysis**

Excised Coomassie Brilliant Blue R250 stained protein spots from the 1D SDS-PAGE gel were chopped into small cubes (approx. 1 mm^3^) and destained with 0.1 M 4-ethylmorpholine acetate (pH 8.1) in 50% acetonitrile (MeCN, v/v). After complete destaining in sonication bath, the gel was washed with water, shrunk by dehydration with acetonitrile and re-swollen in water two times. Next, the pieces of gel were partly dried using a SpeedVac concentrator and then reconstituted with cleavage buffer containing 50 mM 4-ethylmorpholine acetate, 10% MeCN and 2.5 ng/μl trypsin endoprotease (modified sequencing grade, Promega, Madison, WI). Protein digestion was carried out overnight at 37 °C. The resulting peptides were extracted using final concentration of 40% MeCN/0.1% trifluoroacetic acid (TFA). After extraction, the peptides were analyzed in parallel, applying liquid chromatography-mass spectrometry (LC-MS, ESI-qTOF) or off-line peptides separation with following matrix-assisted laser desorption/ionization time-of-flight (MALDI-TOF) MS analysis.

For LC-MS analysis, the volume of resulting peptides was adjusted by 0.1% formic acid (FA, v/v) to decrease the MeCN concentration to 4%, and then 10 µl of the sample was injected into the LC-MS system using a desalting column (Acclaim 300, C18, 3µm, Guard Cartridge, 2.1 x 10mm, Thermo Fisher Scientific, Waltham, MA) at a flow rate of 200 µl/min (Shimadzu, Kyoto, Japan) of 0.1% FA and a separation column (Acclaim 300, C18, 3 µm analytical 2.1 x 150 mm, Thermo Fisher Scientific, Waltham, MA) at a flow rate of 200 µl/min (Ultimate 3000, Thermo Fisher Scientific, Waltham, MA) of water/MeCN (MS grade, Merck, Darmstadt, Germany) gradient: 0-5 min 5% MeCN/0.1% FA; 35 min 50% MeCN/0.1% FA; 40 min 95% MeCN/0.1% FA; 40-45 min 95% MeCN/0.1% FA. A LC column was directly connected to a mass analyzer. The MS analysis was performed on a commercial quadrupole-Time of Flight (qTOF) maXis PLUS MS instrument (Bruker Daltonics, Bremen, Germany) equipped with electrospray ion source (ESI). Mass spectra were obtained in the positive ion mode within an m/z range of 250-2000, MS acquisition time was 50 min and one MS spectrum followed the four MS/MS experiments of the most intensive signals (after two performed MS/MS experiments for each *m/z* signal the value was included in exclusion list for 2 min). The instrument was externally calibrated using Agilent tuning mix, which results in typical mass accuracy below 5 ppm. After the analysis, the spectra were processed using Data Analysis v4.2 software package (Bruker Daltonics, Bremen, Germany).

For MALDI-TOF/TOF analysis, the resulting peptides were directly 10 times diluted in 0.1% TFA (v/v) and subjected to R3 home-made micro-column (Applied Bioscience, Foster City, CA) pre-equilibrated with 0.1% TFA. After desalting with same TFA solution, the peptides were directly eluted from the R3 micro-column with a step gradient of MeCN/0.1% TFA (10, 20, 40 and 80%, v/v) on the MALDI target and the droplets were allowed to dry at ambient temperature and over-laid by 5 mg/ml solution of α-cyano-4-hydroxy-cinnamic acid in 40% MeCN/0.1% TFA. Mass spectra were measured on a MALDI-TOF/TOF mass spectrometer ultraFLEX III (Bruker Daltonics, Bremen, Germany) in reflectron mode equipped with a nitrogen laser (337 nm). MS spectra were calibrated externally using the monoisotopic [M+H]^+^ ion of peptide standards PepMix II (Bruker Daltonics, Bremen, Germany). The positive MALDI-TOF spectra and MS/MS LIFT spectra of selected *m/z* signals were collected in reflectron mode to identify peptide modification by fatty acids. MALDI-TOF MS and MS/MS spectra were interpreted manually.

**Planar lipid bilayers**

Measurements on planar lipid bilayers were performed in Teflon cells separated by a diaphragm with a circular hole (diameter 0.5 mm) bearing the membrane. The proRtxA and RtxA proteins were diluted in TU buffer and added into the grounded cis compartment with a positive potential. The membrane was formed by the painting method using soybean lecithin in n-decane–butanol (9:1, vol/vol). Both compartments contained 150 mM KCl, 10 mM Tris-HCl (pH 7.4), and 2 mM CaCl_2_, the temperature was 25 °C. The membrane current was registered by Ag/AgCl electrodes (Theta) with salt bridges (applied voltage, 50 mV), amplified by LCA-200-100G, LCA-200-10G and LCA-4K-1G amplifiers (Femto), and digitized by use of a KPCI-3108 card (Keithly). For lifetime determination, approximately 400 of individual pore openings were recorded and the dwell times were determined using QuB software with 10 Hz low-pass filter. The kernel density estimation was fitted with a double-exponential function using Gnuplot software. The relevant model was selected by the χ2 value. The error estimates of lifetimes were obtained by the bootstrap analysis. The overall membrane activity was calculated as (I_t_ - I_t0_)/t [pA/s]; I_t_, the membrane current determined after incubation of the membrane with proRtxA or RtxA for time t; I_t0_, the membrane current determined before the addition of proRtxA or RtxA.

**Liposome preparation for surface plasmon resonance (SPR)**

Concentrated liposome solutions of POPC (100%) and POPC/cholesterol (90/10 mol %) were prepared by mixing the required amounts of POPC and cholesterol, dissolved in chloroform to a glass vial. The film was dried under nitrogen gas, and any residual chloroform was removed by placing the films in a vacuum chamber overnight. The dried film was hydrated in 1 ml of inlet buffer (50 mM Tris-HCl, 5 mM imidazole, 1.3 M urea) and extruded through a polycarbonate membrane with 100-nm pores (GE Healthcare BioSciences, Pittsburgh, PA) using a LiposoFast extruder (Avestin Inc., Ottawa, ON) to make large unilamellar vesicles (LUVs). The LUVs were diluted with 1 ml of inlet buffer to make a 5 mM liposome suspension.

**SPR of proRtxA and RtxA**

A sensor chip with a hydrophobic surface (Nicoya Lifesciences, Kitchener, ON) was coated with 100 µl of 5 mM liposome solution at a flowrate of 20 µl/min on an OpenSPR system (Nicoya Lifesciences, Kitchener, ON). To block any non-specific binding, 1% bovine serum albumin (BSA) in inlet buffer was run through at the same flowrate and volume. The proRtxA and RtxA proteins, initially hydrated in TIU buffer (50 mM Tris-HCl, 5 mM imidazole, 8 M urea) for stability, were diluted immediately before injection in TI buffer (50 mM Tris-HCl, 5 mM imidazole). The final buffer concentration of the protein solution was the same as the inlet buffer. All buffers were filtered and degassed before use. Each protein was diluted to concentrations of 27 nM, 9 nM, and 3 nM. After protein injection, inlet buffer was injected so that both association (k_a_) and dissociation rates (k_d_) could be determined. The kinetic evaluation of the binding of each protein to the cholesterol-containing membrane was conducted using the TraceDrawer program. The equilibrium dissociation constant (K_D_) was calculated as K_D_ = k_d_/k_a_.

**Enzyme-linked immunosorbent assay (ELISA)**

Wells of the MaxiSorp ELISA plates (Nunc, Roskilde, Denmark) were coated overnight at 4 °C with 100 μl of cholesterol covalently linked to BSA (Cloud-Clone Corp., Katy, USA) or free BSA (Albumin fraction V, Carl Roth GmbH, Karlsruhe, Germany), each at a concentration of 10 μg/ml in 0.1 M sodium carbonate buffer (pH 9.6). After washing with phosphate-buffered saline (PBS, pH 7.4), the plates were blocked for 2 h at 25 °C with 200 μl of PBS containing 1% BSA and 0.05% Tween-20 (BSA-PBST) and washed with PBS. The coated wells were incubated for 30 min at 25 °C with 100 μl of increasing concentrations of RtxA (0, 1, 5 and 10 μg/ml) diluted in BSA-PBST. After washing with PBS, the wells were incubated for 2 h at 37 °C with 100 μl of mouse monoclonal antibody (mAb) 9D4 (a kind gift of E. Hewlett, University of Virginia, Charlottesville, VA) diluted 1:5000 in BSA-PBST, washed with PBS and incubated for 1 h at 37 °C with 100 μl of horseradish peroxidase-conjugated swine anti-mouse immunoglobulin G diluted 1:1000 in BSA-PBST. After final washing with PBS, o-phenylenediamine peroxidase substrate was added and the plates were incubated at room temperature in the dark. The reaction was stopped by the addition of 50 μl of 2 M H_2_SO_4_ and absorbance at 492 nm was measured.


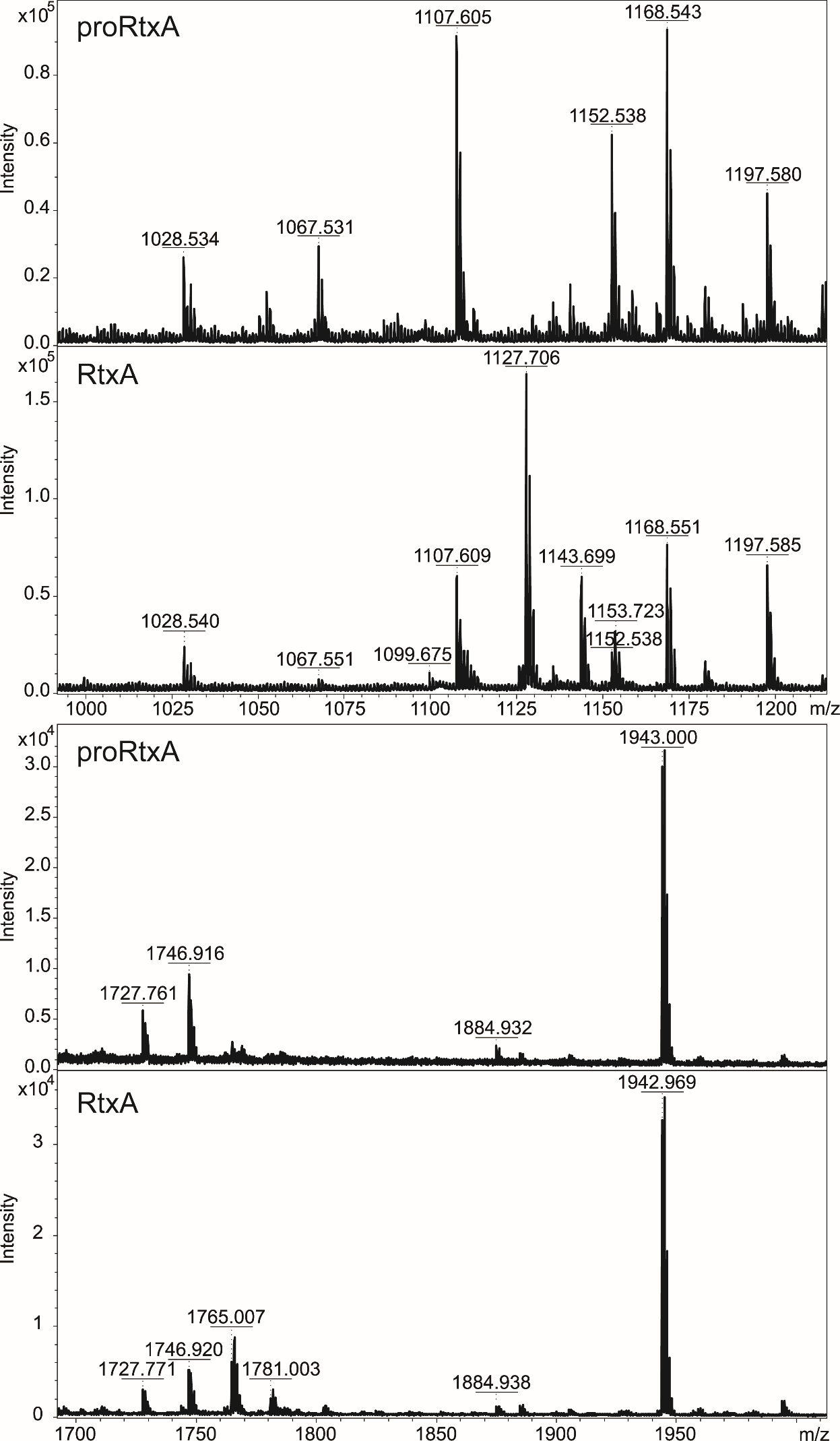


**Supplementary Fig. 1 MS spectra of trypsin-digested proRtxA and RtxA in selected *m/z* ranges.** The tryptic peptides with the m/z signals 1099.675, 1127.706, 1143.699, 1153.723, 1765.007 and 1781.003, which were exclusively present in trypsin-digested RtxA, were further analyzed using a tandem MS/MS sequencing approach.


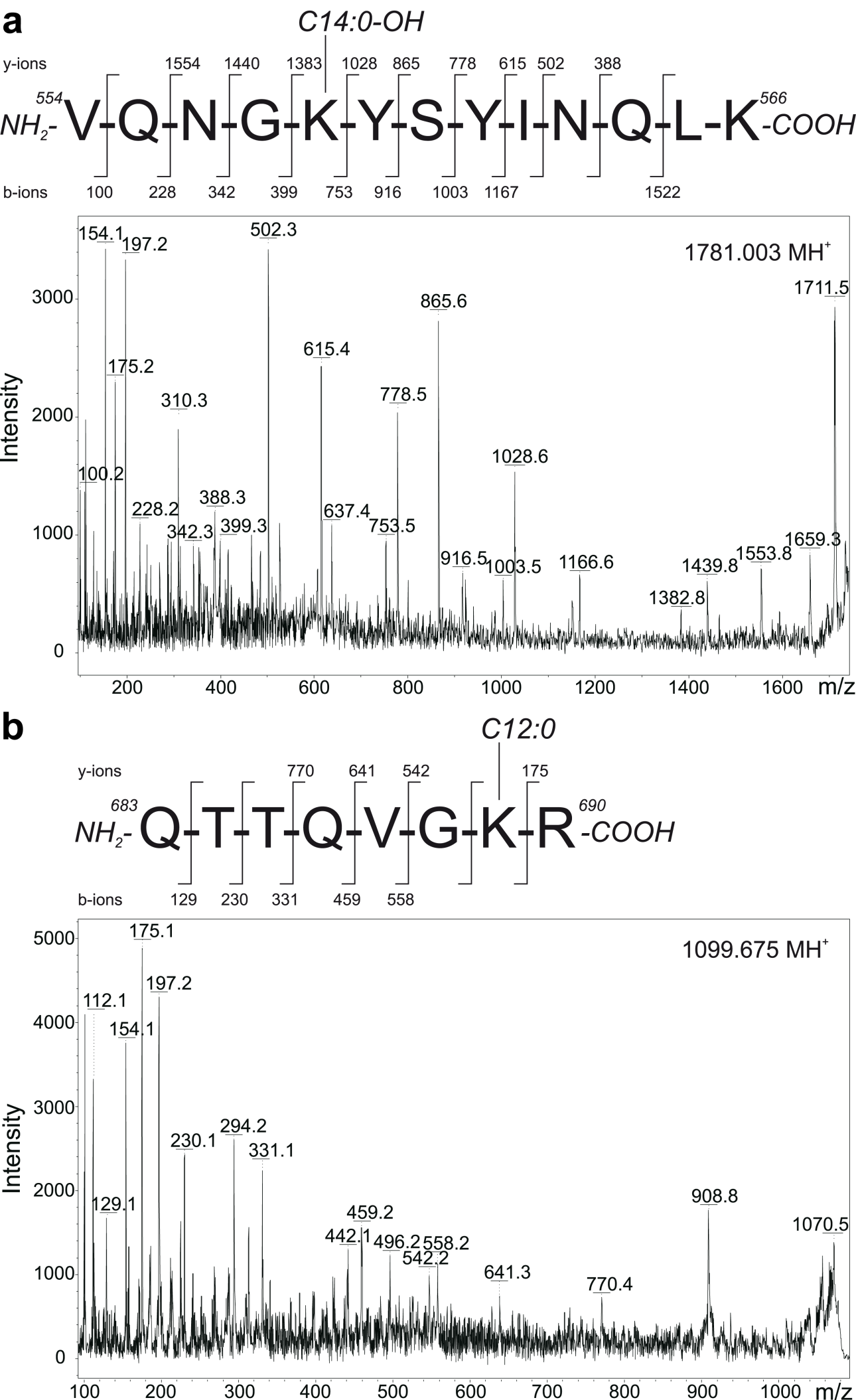


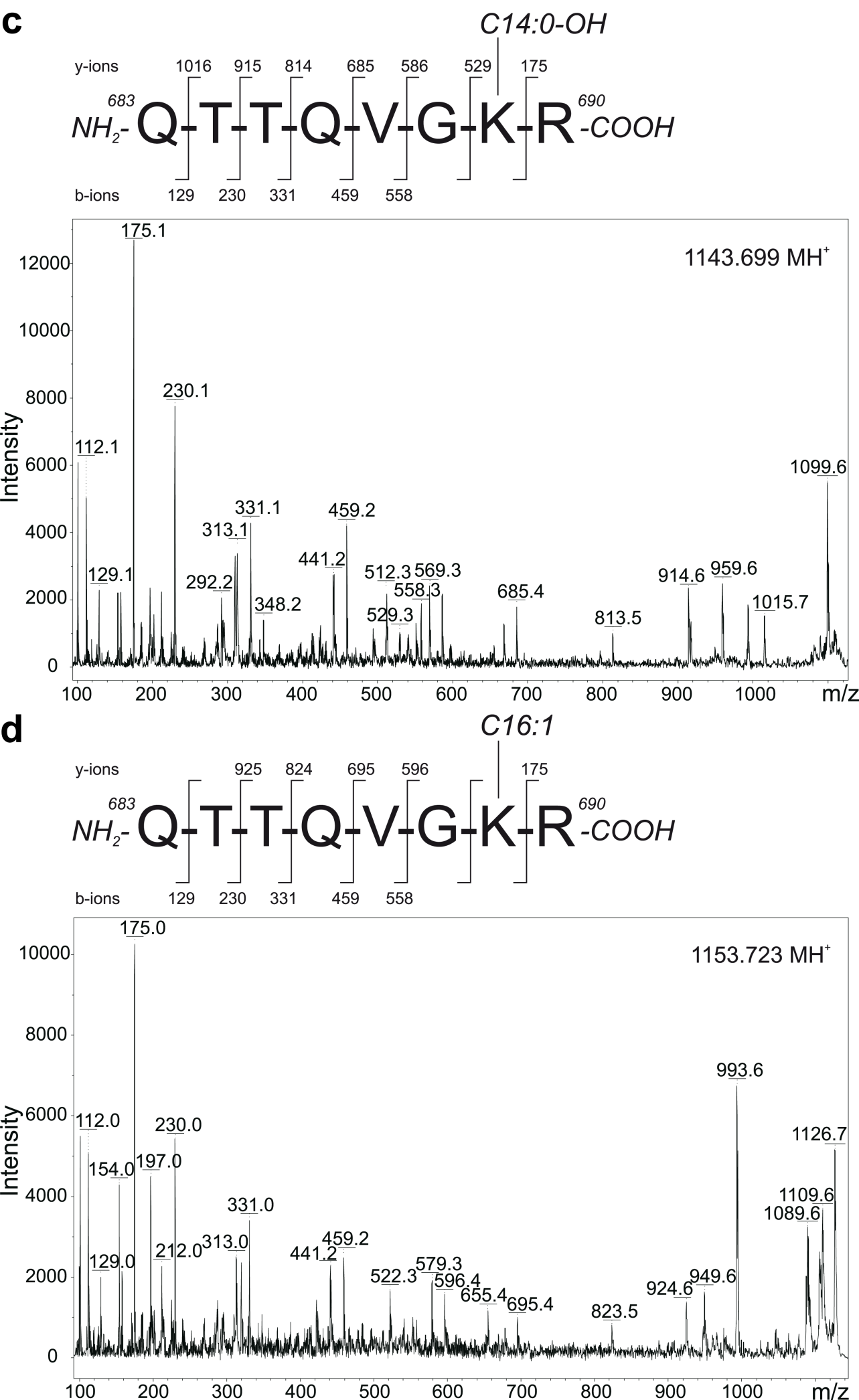


**Supplementary Fig. 2 RtxA is acylated at lysine residues 558 and 689. a** The MS/MS spectrum of the peptide 1781.003 contains b- and y-ions that correspond to the sequence 554-VQNGKYSYINQLK-566 with the ε-amino group of K558 modified by a hydroxymyristoyl (C14:0-OH) acyl chain. **b** The MS/MS spectrum of the peptide 1099.675 contains b- and y-ions that correspond to the sequence 683-QTTQVGKR-690 with a lauroyl (C12:0) acyl group attached to the ε-amino group of K689. **c** The MS/MS spectrum of the peptide 1143.699 contains b- and y-ions that correspond to the sequence 683-QTTQVGKR-690 with the ε-amino group of K689 modified by a hydroxymyristoyl (C14:0-OH) acyl chain. **d** The MS/MS spectrum of the peptide 1153.723 contains b- and y-ions that correspond to the sequence 683-QTTQVGKR-690 with a palmitoleyl (C16:1) acyl group attached to the ε-amino group of K689. The residue numbering corresponds to that of the full-length sequence of the RtxA variant from the *K. kingae* isolate PYKK081.


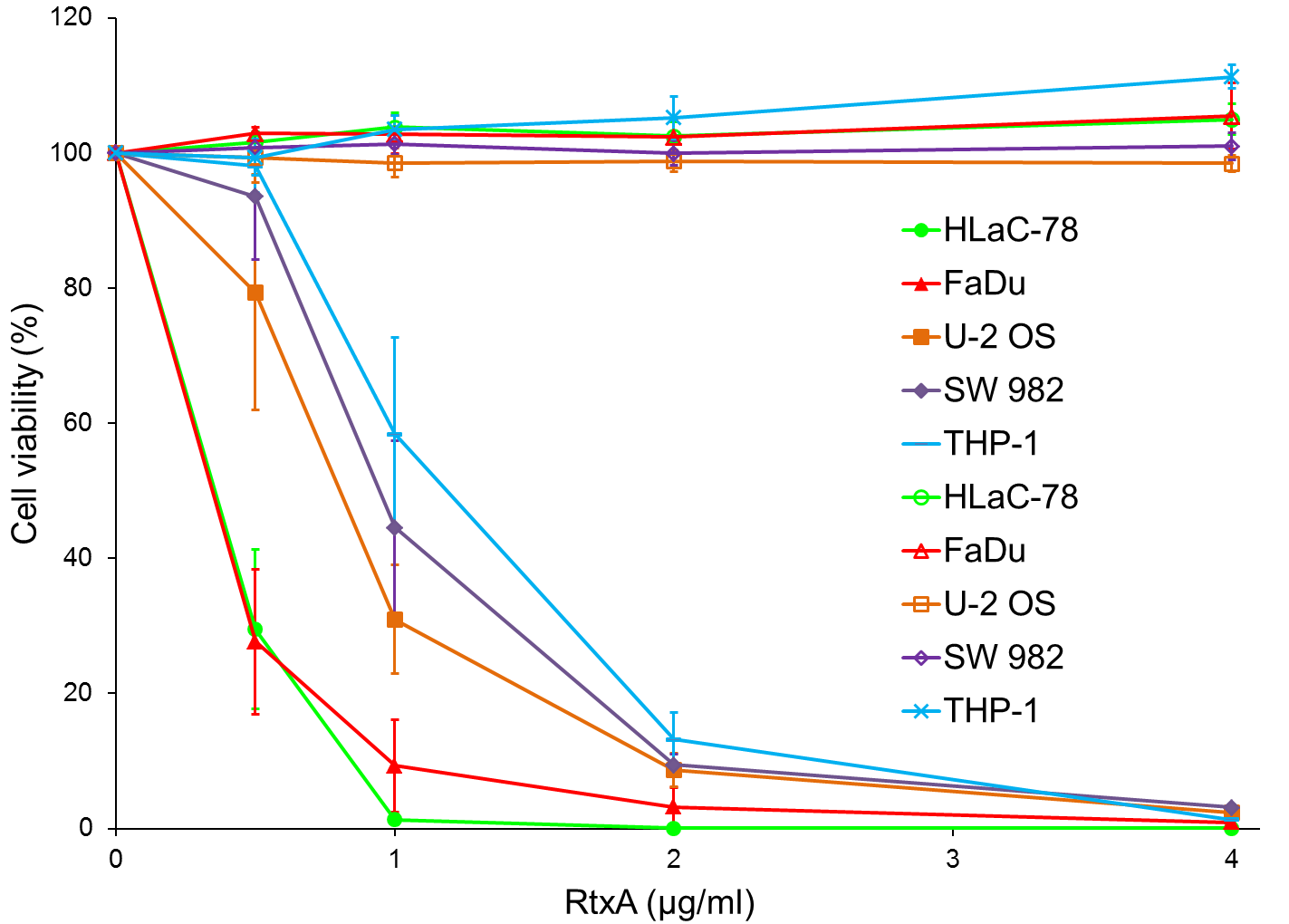


+Ca^2+^

-Ca^2+^

**Supplementary Fig. 3 Cytotoxic activity of RtxA depends on calcium ions.** Different cell types (1×10^6^/ml) were incubated in the absence or in the presence of 2 mM calcium ions with increasing concentrations (0.5, 1, 2 and 4 µg/ml) of purified RtxA for 1 hour at 37 °C. Cell viability was determined by a vital dye staining using 1 µg/ml of Hoechst 33258 followed by flow cytometry. Viability of cells incubated without RtxA was taken as 100%. Each point represents the mean value ± SD of four independent experiments.

**Supplementary Table 1 Confirmation of RtxA identity by peptide mass mapping**

| **Peptide position in RtxA (residues)** | **Experimental**  ***m/z^a^*** | **Theoretical**  ***m/z^b^*** | **Error**  **ppm** |
| --- | --- | --- | --- |
| 462-467 | 745.380 | 745.3844 | 6 |
| 691-697 | 911.448 | 911.4474 | 1 |
| 68-76 | 915.487 | 915.4900 | 3 |
| 445-451 | 925.457 | 925.4644 | 8 |
| 468-475 | 928.548 | 928.5580 | 11 |
| 559-566 | 1028.540 | 1028.5417 | 2 |
| 224-232 | 1030.560 | 1030.5686 | 8 |
| 690-697 | 1067.551 | 1067.5485 | 2 |
| 683-690-C12:0*^c^* | 1099.675 | 1099.6840 | 8 |
| 281-290 | 1107.609 | 1107.5686 | 7 |
| 683-690-C14:0*^c^* | 1127.706 | 1127.7153 | 8 |
| 683-690-C14:0-OH*^c^* | 1143.699 | 1143.7102 | 10 |
| 410-419 | 1152.538 | 1152.5472 | 8 |
| 683-690-C16:1*^c^* | 1153.723 | 1153.7309 | 7 |
| 629-639 | 1158.485 | 1158.4962 | 10 |
| 658-668 | 1168.551 | 1168.5598 | 8 |
| 922-932 | 1197.585 | 1197.5938 | 7 |
| 424-434 | 1244.664 | 1244.6639 | 0 |
| 435-444 | 1248.520 | 1248.5285 | 7 |
| 933-945 | 1357.670 | 1357.6864 | 12 |
| 508-520 | 1375.697 | 1375.7181 | 15 |
| 872-883 | 1382.627 | 1382.7320 | 12 |
| 476-488 | 1387.709 | 1387.7222 | 10 |
| 97-110 | 1421.782 | 1421.8004 | 13 |
| 975-986 | 1464.640 | 1464.6535 | 9 |
| 589-601 | 1499.713 | 1499.7243 | 8 |
| 669-681 | 1530.748 | 1530.7705 | 15 |
| 455-467 | 1542.835 | 1542.8491 | 9 |
| 703-715 | 1545.757 | 1545.7661 | 6 |
| 341-354 | 1633.734 | 1633.7498 | 10 |
| 812-826 | 1663.837 | 1663.8543 | 10 |
| 716-730 | 1672.790 | 1672.8070 | 10 |
| 669-682 | 1686.860 | 1686.8716 | 7 |
| 690-702 | 1699.849 | 1699.8656 | 10 |
| 835-849 | 1727.771 | 1727.7811 | 6 |
| 264-280 | 1746.920 | 1746.9390 | 11 |
| 340-354 | 1761.831 | 1761.8448 | 8 |
| 554-566-C14:0*^c^* | 1765.007 | 1765.0264 | 11 |
| 554-566-C14:0-OH*^c^* | 1781.003 | 1781.0213 | 10 |
| 589-605 | 1884.938 | 1884.9568 | 10 |
| 119-137 | 1942.969 | 1942.9722 | 2 |
| 854-871 | 2043.985 | 2044.0100 | 12 |
| 533-551 | 2072.086 | 2072.1140 | 14 |
| 698-715 | 2178.061 | 2178.0831 | 10 |
| 887-906 | 2202.106 | 2202.1366 | 14 |
| 68-88 | 2228.077 | 2228.1159 | 17 |
| 756-778 | 2306.009 | 2306.0325 | 10 |
| 606-628 | 2320.077 | 2320.1057 | 12 |
| 734-755 | 2336.943 | 2336.9656 | 10 |
| 233-256 | 2378.239 | 2378.2641 | 11 |
| 498-520 | 2443.160 | 2443.1742 | 6 |
| 424-444 | 2474.155 | 2474.1741 | 8 |
| 141-166 | 2704.472 | 2704.4231 | 18 |
| 946-971 | 2999.558 | 2999.4526 | 35 |
| 691-715 | 3070.631 | 3070.5122 | 39 |
| 178-206 | 3160.721 | 3160.6126 | 34 |
| 797-826 | 3225.568 | 3225.4823 | 27 |
| 796-826 | 3353.678 | 3353.5774 | 30 |
| 756-795 | 4052.154 | 4051.8294 | 25 |
| 589-628 | 4186.153 | 4186.0442 | 26 |

*^a^*RtxA purified from *E. coli* was separated by SDS-PAGE, the most abundant protein band with an apparent size of 105 kDa was excised from the gel, subjected to in-gel trypsin digestion and the *m/z* values of the extracted peptides were determined by MALDI-TOF MS analysis.

*^b^*The *m/z* values computed for RtxA peptide fragments expected to result from trypsin digestion of RtxA of the *K. kingae* isolate PYKK081.

*^c^*The peptide fragments with covalently linked acyl chains: C12:0, lauric acid; C14:0, myristic acid; C14:0-OH, hydroxylated myristic acid; C16:1, palmitoleic acid.

**Supplementary Table 2 Confirmation of proRtxA identity by peptide mass mapping**

| **Peptide position in proRtxA** | **Experimental**  ***m/z^a^*** | **Theoretical**  ***m/z***^b^ | **Error**  **ppm** |
| --- | --- | --- | --- |
| 462-467 | 745.375 | 745.3844 | 13 |
| 691-697 | 911.435 | 911.4474 | 14 |
| 68-76 | 915.483 | 915.4900 | 8 |
| 445-451 | 925.456 | 925.4644 | 9 |
| 468-475 | 928.543 | 928.5580 | 16 |
| 224-232 | 1030.544 | 1030.5686 | 24 |
| 690-697 | 1067.531 | 1067.5485 | 16 |
| 281-290 | 1107.605 | 1107.5686 | 10 |
| 410-419 | 1152.538 | 1152.5472 | 8 |
| 629-639 | 1158.492 | 1158.4962 | 4 |
| 658-668 | 1168.543 | 1168.5598 | 14 |
| 922-932 | 1197.58 | 1197.5938 | 12 |
| 424-434 | 1244.641 | 1244.6639 | 18 |
| 435-444 | 1248.514 | 1248.5285 | 12 |
| 933-945 | 1357.664 | 1357.6864 | 17 |
| 508-520 | 1375.71 | 1375.7181 | 6 |
| 872-883 | 1382.630 | 1382.7320 | 10 |
| 476-488 | 1387.701 | 1387.7222 | 15 |
| 97-110 | 1421.775 | 1421.8004 | 18 |
| 975-986 | 1464.632 | 1464.6535 | 15 |
| 589-601 | 1499.704 | 1499.7243 | 14 |
| 669-681 | 1530.751 | 1530.7705 | 13 |
| 455-467 | 1542.834 | 1542.8491 | 10 |
| 703-715 | 1545.747 | 1545.7661 | 12 |
| 341-354 | 1633.734 | 1633.7498 | 10 |
| 812-826 | 1663.834 | 1663.8543 | 12 |
| 716-730 | 1672.792 | 1672.8070 | 9 |
| 669-682 | 1686.849 | 1686.8716 | 13 |
| 690-702 | 1699.84 | 1699.8656 | 15 |
| 835-849 | 1727.761 | 1727.7811 | 12 |
| 264-280 | 1746.916 | 1746.9390 | 13 |
| 340-354 | 1761.820 | 1761.8448 | 14 |
| 589-605 | 1884.932 | 1884.9568 | 13 |
| 119-137 | 1943.000 | 1942.9722 | 10 |
| 854-871 | 2043.977 | 2044.0100 | 16 |
| 533-551 | 2072.085 | 2072.1140 | 14 |
| 698-715 | 2178.053 | 2178.0831 | 14 |
| 887-906 | 2202.101 | 2202.1366 | 16 |
| 68-88 | 2228.096 | 2228.1159 | 9 |
| 756-778 | 2305.998 | 2306.0325 | 15 |
| 606-628 | 2320.074 | 2320.1057 | 14 |
| 734-755 | 2336.947 | 2336.9656 | 8 |
| 233-256 | 2378.236 | 2378.2641 | 12 |
| 498-520 | 2443.146 | 2443.1742 | 12 |
| 424-444 | 2474.145 | 2474.1741 | 12 |
| 141-166 | 2704.426 | 2704.4231 | 1 |
| 946-971 | 2999.491 | 2999.4526 | 13 |
| 691-715 | 3070.545 | 3070.5122 | 11 |
| 178-206 | 3160.679 | 3160.6126 | 21 |
| 797-826 | 3225.574 | 3225.4823 | 28 |
| 796-826 | 3353.607 | 3353.5774 | 9 |
| 756-795 | 4051.949 | 4051.8294 | 29 |
| 589-628 | 4186.145 | 4186.0442 | 24 |

*^a^*proRtxA purified from *E. coli* was separated by SDS-PAGE, the most abundant protein band with an apparent size of 105 kDa was excised from the gel, subjected to in-gel trypsin digestion and the *m/z* values of the extracted peptides were determined by MALDI-TOF MS analysis.

*^b^*The *m/z* values computed for proRtxA peptide fragments expected to result from trypsin digestion of proRtxA of the *K. kingae* isolate PYKK081.

**Supplementary Table 3 Acylation status of the Lys558 and Lys689 residues of RtxA**

| **Residue*^a^*** | **Acyl chain*^a^*** | **m/z**^b^ | **Relative distribution of acyl chains*^c^* (%)** |
| --- | --- | --- | --- |
| K558 | C14:0 | 1765.007 | 17.9 ± 9.0 |
| K558 | C14:0-OH | 1781.003 | 4.9 ± 1.4 |
| K689 | C12:0 | 1099.675 | 2.2 ± 0.9 |
| K689 | C14:0 | 1127.706 | 71.3 ± 1.9 |
| K689 | C14:0-OH | 1143.699 | 17.5 ± 2.3 |
| K689 | C16:1 | 1153.723 | 8.0 ± 3.5 |

*^a^*RtxA was produced in the presence of the RtxC acyltransferase in *E. coli* BL21/pMM100 cells, purified close to homogeneity and analyzed by MS. The ε-amino groups of the K558 and K689 residues were found to be modified by lauroyl (C12:0), myristoyl (C14:0), hydroxymyristoyl (C14:0-OH) and/or palmitoleyl (C16:1) chains.

*^b^*The m/z values of the 554-VQNGKYSYINQLK-566 and 683-QTTQVGKR-690 peptides of RtxA covalently modified with acyl chains.

*^c^*Percentage distribution of acyl chain modification of the ε-amino groups of the K558 and K689 residues of RtxA. Average values of three determinations ± standard deviations. The remaining K558 and K689 residues to 100% are non-acylated.

**Supplementary Table 4** **Kinetic analysis of RtxA and proRtxA binding to POPC and POPC/cholesterol membranes**

| **Sample*^a^*** | **k_a_ (1/M·s)*^b^*** | **k_d_ (1/s)*^b^*** | **K_D_ (M)*^c^*** |
| --- | --- | --- | --- |
| RtxA + POPC | 2.72 ( ± 0.45) x 10^5^ | 4.10 ( ± 2.31) x 10^-4^ | 1.46 ( ± 0.68) x 10^-9^ |
| RtxA + POPC/cholesterol | 2.32 ( ± 0.56) x 10^5^ | 4.42 ( ± 5.29) x 10^-5^ | 1.71 ( ± 1.87) x 10^-10^ |
| proRtxA + POPC | 4.19 ( ± 0.71) x 10^5^ | 4.70 ( ± 0.14) x 10^-4^ | 1.14 ( ± 0.16) x 10^-9^ |
| proRtxA + POPC/cholesterol | 3.39 ( ± 0.76) x 10^5^ | 2.71 ( ± 2.03) x 10^-4^ | 7.39 ( ± 4.04) x 10^-10^ |

***^a^***A sensor chip of an OpenSPR system was coated with liposomes made of POPC (100%) and POPC/cholesterol (90/10 mol %). The RtxA and proRtxA proteins were passed over the chip surface at concentrations 27, 9 and 3 nM.

***^b^***The kinetic evaluation of the binding of each protein to the membranes was conducted using the TraceDrawer program.

***^c^***The equilibrium dissociation constant (K_D_) was calculated as K_D_ = k_d_/k_a_.

**Supplementary Table 5:** Predicted CRAC and CARC motifs in the pore-forming domain of RtxA

| **RtxA residues** | **Cholesterol recognition motif^a^** | **Sequences of the predicted CRAC and CARC motifs^b^** |
| --- | --- | --- |
| 48-58 | CRAC | LTIPKD**Y**DIEK |
| 280-287 | CARC | KAISS**Y**VL |
| 340-348 | CARC | KFG**Y**DGDSL |
| 349-354 | CRAC | LAE**Y**QR |
| 444-453 | CARC | RHAH**Y**LERNL |

^a^CRAC motif has the consensus sequence L/V-(X)(1–5)-Y-(X)(1–5)-R/K and CARC motif consists of the R/K-(X)(1–5)-Y/F-(X)(1–5)-L/V) pattern, where (X)(1–5) represents between one and five residues of any amino acid.

^b^The central tyrosine residues of the CRAC/CARC motifs are shown in bold.
